# Inter-society consensus for the use of inhaled corticosteroids in infants, children and adolescents with airway diseases

**DOI:** 10.1186/s13052-021-01013-8

**Published:** 2021-04-21

**Authors:** Marzia Duse, Francesca Santamaria, Maria Carmen Verga, Marcello Bergamini, Giovanni Simeone, Lucia Leonardi, Giovanna Tezza, Annamaria Bianchi, Annalisa Capuano, Fabio Cardinale, Giovanni Cerimoniale, Massimo Landi, Monica Malventano, Mariangela Tosca, Attilio Varricchio, Anna Maria Zicari, Carlo Alfaro, Salvatore Barberi, Paolo Becherucci, Roberto Bernardini, Paolo Biasci, Carlo Caffarelli, Valeria Caldarelli, Carlo Capristo, Serenella Castronuovo, Elena Chiappini, Renato Cutrera, Giovanna De Castro, Luca De Franciscis, Fabio Decimo, Iride Dello Iacono, Lucia Diaferio, Maria Elisa Di Cicco, Caterina Di Mauro, Cristina Di Mauro, Dora Di Mauro, Francesco Di Mauro, Gabriella Di Mauro, Mattia Doria, Raffaele Falsaperla, Valentina Ferraro, Vassilios Fanos, Elena Galli, Daniele Giovanni Ghiglioni, Luciana Indinnimeo, Ahmad Kantar, Adima Lamborghini, Amelia Licari, Riccardo Lubrano, Stefano Luciani, Francesco Macrì, Gianluigi Marseglia, Alberto Giuseppe Martelli, Luigi Masini, Fabio Midulla, Domenico Minasi, Vito Leonardo Miniello, Michele Miraglia del Giudice, Sergio Renzo Morandini, Germana Nardini, Agostino Nocerino, Elio Novembre, Giovanni Battista Pajno, Francesco Paravati, Giorgio Piacentini, Cristina Piersantelli, Gabriella Pozzobon, Giampaolo Ricci, Valter Spanevello, Renato Turra, Stefania Zanconato, Melissa Borrelli, Alberto Villani, Giovanni Corsello, Giuseppe Di Mauro, Diego Peroni

**Affiliations:** 1 Department of Pediatrics, Policlinico Umberto I, Sapienza University of Rome, Rome, Italy; 2 Department of Translational Medical Sciences, Federico II University, Naples, Italy; 3 Family Pediatrician Local Health Unit Salerno, Vietri sul Mare, Italy; 4 Family Pediatrician, Local Health Unit, Ferrara, Italy; 5 Family Pediatrician, Local Health Unit, Mesagne, Italy; 6 Maternal, Infantile and Urological Sciences Department, Sapienza University, Rome, Italy; 7 Pediatric Department, Franz Tappeiner Hospital, Meran, Italy; 8 Pediatric Unit, Department of Women’s and Children’s Health, San Camillo Forlanini Hospital, Rome, Italy; 9 Department of Experimental Medicine, University “Luigi Vanvitelli”, Regional Centre of Pharmacovigilance Campania, Naples, Italy; 10 Pediatric and Emergency Unit Giovanni XXIII Pediatric Hospital University of Bari, Bari, Italy; 11 Family Pediatrician Local Health Unit Latina, Latina, Italy; 12 Family Pediatrician Local Health Unit, Turin and IRIB-CNR, Palermo, Italy; 13 Family Pediatrician Local Health Unit, Ferrara, Italy; 14 Allergy Centre, Department of Pediatric Sciences IRCCS Gaslini Institute, Genova, Italy; 15 Departmental Operative Unit of Diagnostic and Surgical Videoendoscopy of the Upper Airways, Asl Napoli 1 Center, Naples, Italy; 16 Maternal, infantile and urological sciences Department, Pediatric Allergic Unit, Sapienza University, Rome, Italy; 17 Paediatrics Unit, Reunited Hospitals Castellammare of Stabia, Naples, Italy; 18 Paediatric Unit, ASST-Rhodense, RHO, Italy; 19 Family Pediatrician, Local Health Unit Tuscany center, Florence, Italy; 20 Pediatric Unit San Giuseppe Hospital, Empoli, Florence, Italy; 21 Family Paediatrician, Local Health Unit, FIMP National President, Livorno, Italy; 22 Department of Obstetrics Gynaecology and Pediatrics, Azienda USL-IRCCS di Reggio Emilia, Reggio Emilia, Italy; 23 Pediatric Unit, Department of Mother and Child, AUSL-IRCCS, Reggio Emilia, Italy; 24 Department of Woman, Child and of General and Specialized Surgery, University “Luigi Vanvitelli”, Naples, Italy; 25 Family Paediatrician Local Health Unit Nettuno-Anzio, Rome, Italy; 26 Paediatric Infectious Disease Unit, Meyer Children’s University Hospital, Department Of Health Sciences, University of Florence, Florence, Italy; 27 Pediatric Pulmonology Unit, Academic Department of Paediatrics, Bambino Gesù Children’s Hospital, IRCCS, Rome, Italy; 28 Endocrinologist Specialist, Salerno, Italy; 29 Independent Paediatrician Researcher, Benevento, Italy; 30 Department of Paediatrics, Aldo Moro University of Bari, Bari, Italy; 31 Paediatrics Unit, University Hospital of Pisa, Department of Clinical and Experimental Medicine, University of Pisa, Pisa, Italy; 32 General Paediatrics and Paediatric Acute and Emergency Unit, University Hospital San Marco, University of Catania, Catania, Italy; 33 Family Paediatrician Local Health Unit, Ausl, Modena, Italy; 34 Family Paediatrician, Local Health Unit, Caserta, Italy; 35 Primary Care Paediatrician, Local Health Unit, National Secretary for the Scientific and Ethical Activities of FIMP, Chioggia, Italy; 36 Neonatal Intensive Care Unit and Neonatal Accompaniment Unit, University Hospital San Marco, University of Catania, Catania, Italy; 37 Unit of Paediatric Allergy and Respiratory Medicine Women’s and Children’s Health Department, University Hospital Padua, Padua, Italy; 38 Neonatal Intensive Care Unit, Neonatal Pathology and Neonatal Section, AOU and University of Cagliari, Monserrato (CA), Italy; 39 Pediatric Allergy Unit, Department of Paediatric Medicine, S. Pietro Hospital Fatebenefratelli, Rome, Italy; 40 Foundation IRCCS Ca’ Granda Ospedale Maggiore Policlinico di Milano, UOSD Paediatric Highly Intensive Care Unit, Milan, Italy; 41 Pediatric Asthma and Cough Center Istituti Ospedalieri Bergamaschi, Gruppo Ospedaliero San Donato, Ponte San Pietro, Bergamo, Italy; 42 Family Pediatrician Local Health Unit Teramo, Teramo, Italy; 43 Paediatric and Neonatology Unit Santa Maria Goretti Hospital, Department of Pediatrics, University of Pavia, Pavia, Italy; 44 Paediatric and Neonatology Unit Santa Maria Goretti Hospital, Department of Pediatrics, Sapienza University, Rome, Italy; 45 Pediatric and Neonatal Intensive Care Unit Fatebenefratelli Isola Tiberina, Rome, Italy; 46 Allergist Pediatrician National Secretary of Italian Federation for Medical Scientific Societies (FISM), Rome, Italy; 47 Department of Pediatrics G. Salvini Hospital Garbagnate Milanese, Milan, Italy; 48 Pediatric Pulmonology and Subintensive Respiratory Therapy Unit Department of Pediatrics Santobono-Pausilipon Children’s Hospital, Naples, Italy; 49 Pediatric Unit Great Metropolitan Hospital Reggio Calabria, Reggio Calabria, Italy; 50 Department of Biomedical Science and Human Oncology, University of Bari, Children’s Hospital “Giovanni XXIII”, Bari, Italy; 51 Family Pediatrician, Local Health Unit, Latina, Italy; 52 Department of Translational Medical Sciences, Pediatric Pulmonology, Federico II University, Naples, Italy; 53 Division of Pediatrics, University Hospital of Udine, Udine, Italy; 54 Department Health Science, University of Florence, Florence, Italy; 55 Department of Human Pathology in Adult and Development Age, Pediatric Unit, University of Messina, Messina, Italy; 56 Pediatric Unit, S. Giovanni di Dio Hospital, Crotone, Italy; 57 Paediatric Section Department of Surgery, Dentistry, Paediatrics and Gynaecology University of Verona, Verona, Italy; 58 Family Pediatrician, Paediatric Allergy, Local Health Unit TO1, Turin, Italy; 59 Department of Pediatrics, IRCC San Raffaele, Milan, Italy; 60 Alma Mater Studiorum, Univesity of Bologna, Bologna, Italy; 61 Family Pediatrician Local Health Unit, Caselle Torinese, Vicenza, Italy; 62 Family Pediatrician Local Health Unit Caselle Torinese, Turin, Italy; 63 Unit of Pediatric Allergy and Respiratory Medicine Women’s and Children’s Health Department University Hospital, Padua, Italy; 64 Family Pediatrician, Local Health Unit Salerno, Aversa, Italy; 65 Department of Clinical and Experimental Medicine, Section of Pediatrics, University of Pisa, Pisa, Italy

**Keywords:** Inhaled corticosteroids, Asthma, Wheezing, Rhinitis, Rhinosinusitis, Laryngospasm, Laryngotracheitis, Adenoid hypertrophy

## Abstract

**Background:**

In 2019, a multidisciplinary panel of experts from eight Italian scientific paediatric societies developed a consensus document for the use of inhaled corticosteroids in the management and prevention of the most common paediatric airways disorders. The aim is to provide healthcare providers with a multidisciplinary document including indications useful in the clinical practice. The consensus document was intended to be addressed to paediatricians who work in the Paediatric Divisions, the Primary Care Services and the Emergency Departments, as well as to Residents or PhD students, paediatric nurses and specialists or consultants in paediatric pulmonology, allergy, infectious diseases, and ear, nose, and throat medicine.

**Methods:**

Clinical questions identifying Population, Intervention(s), Comparison and Outcome(s) were addressed by methodologists and a general agreement on the topics and the strength of the recommendations (according to the GRADE system) was obtained following the Delphi method. The literature selection included secondary sources such as evidence-based guidelines and systematic reviews and was integrated with primary studies subsequently published.

**Results:**

The expert panel provided a number of recommendations on the use of inhaled corticosteroids in preschool wheezing, bronchial asthma, allergic and non-allergic rhinitis, acute and chronic rhinosinusitis, adenoid hypertrophy, laryngitis and laryngospasm.

**Conclusions:**

We provided a multidisciplinary update on the current recommendations for the management and prevention of the most common paediatric airways disorders requiring inhaled corticosteroids, in order to share useful indications, identify gaps in knowledge and drive future research.

## Background and aim of the document

In 2019, the Italian Society for Paediatrics (SIP), the Italian Society of Paediatric Respiratory Diseases (SIMRI), the Italian Society for Paediatric Allergy and Immunology (SIAIP), the Italian Society for Preventive and Social Paediatrics (SIPPS), the Italian Society of Paediatric Primary Care (SICuPP), the Italian Society of Adolescent Medicine (SIMA), the Italian Society of Paediatric Emergency Medicine (SIMEUP) and the Italian Federation of Paediatricians (FIMP) provided an update on the available recommendations for the use of inhaled corticosteroids (ICS) in the management and prevention of the most common paediatric airways disorders. Intersociety documents are currently considered the most effective tool to reach all healthcare providers, to convey shared indications useful in the clinical practice, to identify any gaps in current knowledge and to drive research.

In order to improve current knowledge on the topic and share indications for future studies, a multidisciplinary panel including emergency and primary care paediatricians and experts in Pulmonology, Allergy, Immunology, Pharmacology, Infectious Diseases, Ear, Nose and Throat (ENT), Emergency Medicine and Methodology was established.

The consensus panel settled the general aims of the project and developed the timing of each phase, topics and methods of consent, consultation, research and selection of the evidence.

The consensus document was intended to be addressed to paediatricians who work in the Paediatric Divisions, the Primary Care Services and the Emergency Departments, as well as to Residents or PhD students, paediatric nurses and specialists or consultants in paediatric ENT, pulmonology, allergy, and infectious diseases.

The use of ICS in the following paediatric airway disorders was considered:

- infant and preschool wheezing;
- bronchial asthma;
- persistent allergic and non-allergic rhinitis;
- acute and chronic rhinosinusitis;
- adenoid hypertrophy;
- laryngitis and laryngospasm.

Clinical questions identifying Population, Intervention (s), Comparison and Outcome(s) (P.I.C.O.) were addressed by methodologists. Finally, a general agreement on the topics and the strength of the recommendations according to the GRADE system was obtained following the Delphi method [1].

The literature selection included secondary sources such as Evidence-based Guidelines (GL) and Systematic Reviews (SR) and was integrated with Primary Studies published after those included in the SRs or GL. Search strategy aimed at gathering studies on immunocompetent paediatric patients with the above mentioned disorders. The GL published in the previous 5 years and/or reviews or SRs published in the previous 10 years were included. No time limit was considered for the primary studies. Studies focusing on genetic disorders, chronic lung disorders, inborn errors of immunity, comorbidities or other risk factors for individual pathologies were excluded. The literature search was completed in December 2018 for persistent allergic and non-allergic rhinitis, in February 2019 for acute and chronic rhinosinusitis, in December 2020 for wheezing and asthma, adenoid hypertrophy and laryngotracheitis/laryngospasm. The following keywords were used:

- *Population*

respiratory sounds, wheezing, sibilant rhonchi, viral wheezing, asthma, rhinitis, rhinosinusitis and acute, chronic,sinusitis, adenotonsillar hypertrophy, enlargement, croup, acute laryngitis, laryngotracheitis, laryngismus

- *Intervention (s), Comparison*

nebulizers and vaporizers, inhalation device, nasal steroids, fluticasone propionate, budesonide, mometasone,beclomethasone,corticosteroids, topical, nasal, adrenal cortex hormones, inhaled corticosteroids,montelukast,leukotriene receptor blocking agent, leukotriene antagonists, histamine h1 antagonists;

- *Outcome(s)*

exacerbations requiring oral corticosteroids, recurrence, symptom-free days, asthma symptom score (Day time, Night time), adverse events, linear growth, medication score, symptom score, complications, hospitalization, emergency medical services, quality of life, malocclusion, adenoidectomy, nasal obstruction, sleep apnea syndromes, obstructive sleep apnea, obstructive sleep apnea syndrome (OSAS), otitis media with effusion, duration of acute illness, rate of access to emergency department, hospital admission rate;

- *Limits*

adolescent, child, preschool child and infant, young child.

At least two authors searched for and evaluated the scientific evidences, independently. In case of discrepancy, the decision was taken via collegial discussion.

The methodologists used the following tools for evaluating the validity of the studies analyzed: the Grilli criteria for Consensus and Position Papers [2], the AGREE II checklist for GL [3], the AMSTAR-2 for the SR [4]. For randomized studies, the risk of bias was assessed using the Cochrane “Risk of bias” tool, while the certainty of the body of evidence was assessed for the primary outcomes using the GRADE approach [5]. The Newcastle Ottawa Scale-checklists were used for observational studies including cohort, case-control and cross-sectional studies [6]. The minimum validity criterion for inclusion was considered the absence of relevant bias.

In order to provide recommendations, an evidence-based critical analysis was used according to the GRADE method [7–9]. Recommendations based on the grading of the quality of the available evidence were provided when possible, and expert consensus was used when data was lacking. The strength of the recommendation was expressed as: Strong or Weak recommendation in favor or against the intervention, and the strength determinants were: desirable and unwanted effects ratio, quality of evidence, patient’s preference, costs (allocation of resources).

## Pharmacology of ICS

**Fig. 1:**
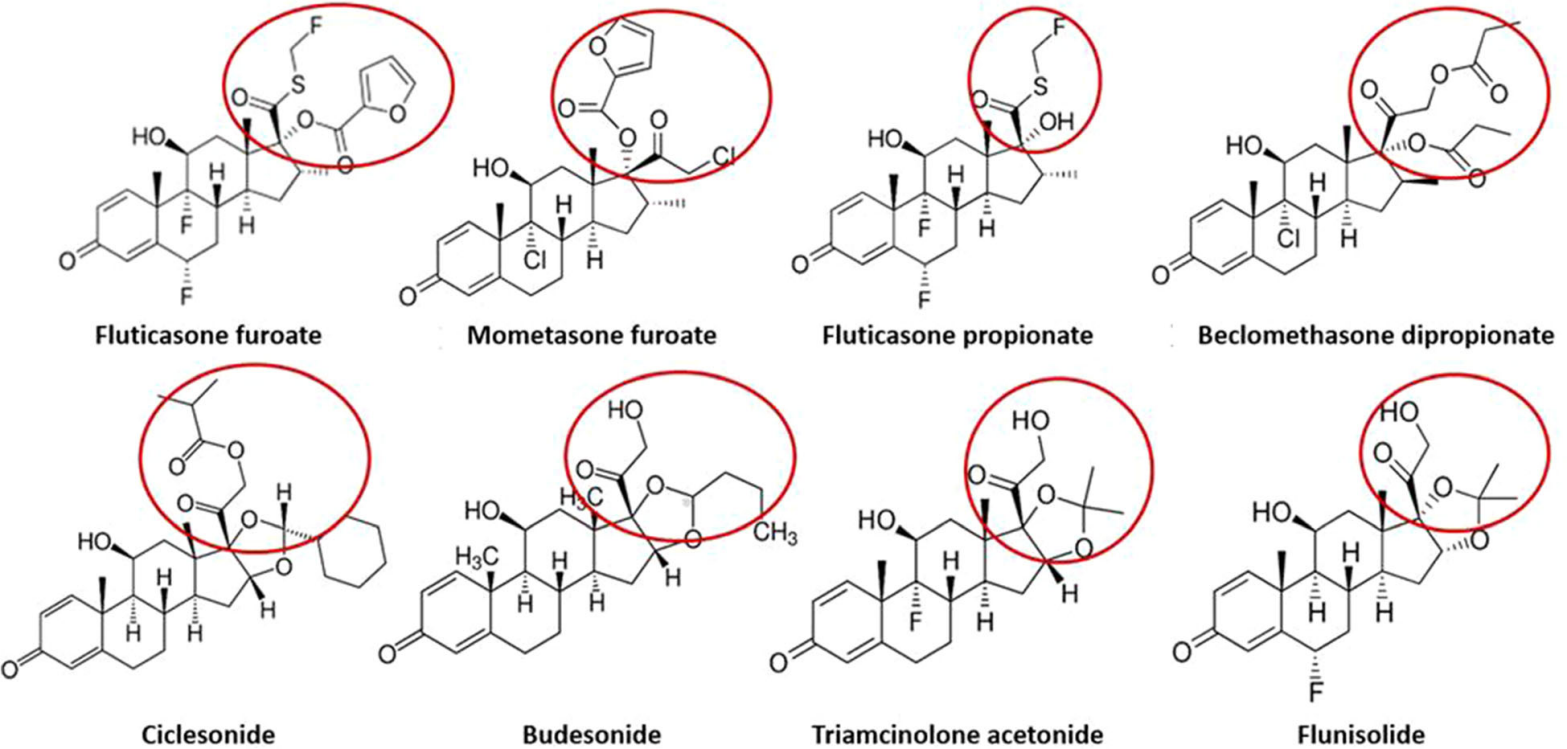
Inhaled corticosteroids currently available

Synthetic glucocorticoids derive from modifications to the chemical structure of cortisol, which is the main glucocorticoid released from the zona fasciculata layer of the adrenal cortex in response to the pituitary release of the AdrenoCorticoTropic Hormone (ACTH). Synthetic glucocorticoids notably have strong anti-inflammatory activity, while mineralocorticoid action is negligible. A remarkable effect of glucocorticoids on the metabolism of carbohydrates, proteins and lipids is well known. In order to allow local administration of these compounds, more lipophilic molecules –via modifications on C-17 and C-21- have been introduced. Eight different ICS are currently available (Fig. 1): *beclomethasone dipropionate*; *budesonide*; *ciclesonide*; *flunisolide*; *fluticasone furoate*; *fluticasone propionate*; *mometasone furoate;* and *triamcinolone acetonide*. The physiological effects of ICSare mediated by a dual mechanism, i.e. ICS regulate either positively or negatively the proteins synthesis in the target cells through a genomic mechanism, and a rapid vasoconstriction in the airways is provided through a faster, non-genomic, mechanism [10].

Multiple factors can significantly affect both pharmacokinetics and pharmacodynamics of ICS, including age-related metabolism, bioavailability, drug-protein binding and lipophilia. The efficiency of the mechanisms of absorption, distribution, metabolism and excretion of ICS significantly changes with ageing [11]. The bioavailability of ICS is different within the respiratory tract (*pulmonary bioavailability*) and in the systemic circulation (*oral bioavailability*). The drug-protein binding affects toxicity and efficacy of ICS. Finally, ICS are classified according to their lipophilia: *fluticasone furoate*> > *mometasone furoate* ≥ *fluticasone proprionate* > *triamcinoloneacetonide*> > *budesonide* ≥ *ciclesonide* > *flunisolide* ≥ *beclomethasone dipropionate* [12].

### Potency, dose equivalence and therapeutic index

The main factors that influence the efficacy of ICS are the potency of the compound, the efficiency of the inhaler device and the drug-target residence time. These factors drive physicians in the choice of the most appropriate dose of ICS. Among the available ICS, those with a longer pulmonary residence require a single daily administration, while ICS with a shorter pulmonary residence time will need to be administered two or three times a day. The potency of ICS is significantly influenced by receptor affinity. However, a greater receptor affinity is not always an advantage, as this is often correlated with greater toxicity [13]. In conclusion, a higher therapeutic index of ICS (therapeutic index = TD50 (toxic dose)/ED50 (effective dose), as reported for ciclesonide, fluticasone furoate, fluticasone propionate and mometasone furoate, is associated with greater potency, drug-target residence time, systemic clearance and low oral bioavailability [12]. Wheezing and asthma represent the most frequent indication for ICS treatment and a recent study reported all approved molecules and their minimum effective dose (MED) in paediatric patients [14].

### Safety profile of ICS

Several clinical trials have demonstrated that the incidence of adverse events, both local and systemic, related to ICS is significantly lower compared to systemic steroids. Nevertheless, adverse events related to ICS administration still represent a matter of concern in children or adolescents. Therefore, the type of ICS to be chosen as well as its correct dose and length of treatment are still largely discussed [15]. Proper management of ICS administration can help minimizing the occurrence of systemic adverse events, [16], while clear and correct information to patients should be always provided.

#### Local adverse events

The most common local adverse events induced by ICS are dry throat, dysphonia and oropharyngeal candidiasis. Respiratory distress can occur more rarely [17]. In order to prevent the risk of adverse events in children, the lower effective dose of ICS should be always used. In addition, washing both mouth and teeth after each inhalation is recommended. With regard to spray formulations, the use of spacers is recommended as well.

#### Systemic adverse events

**Fig. 2:**
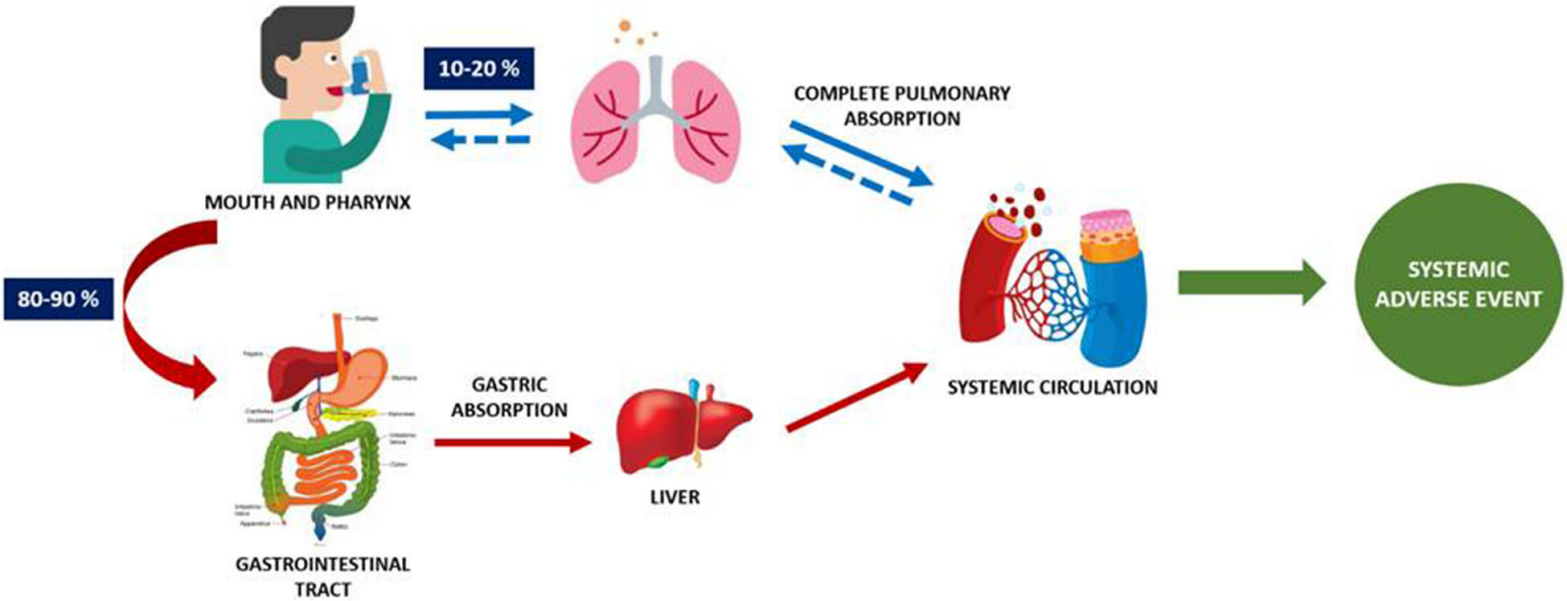
“Pulmonary” bioavailability and “oral” bioavailability of inhaled corticosteroids

ICS can rarely cause also systemic adverse events (Fig. 2), mostly related to the hypothalamic-pituitary-adrenal axis suppression with effects on growth, alteration of bone and finally impairment of carbohydrate metabolism. However, literature data related to the effects of ICS on HPA axis suppression are still conflicting due to the diversity of experimental trial designs [18]. Corticosteroids induce HPA axis suppression by improving negative feedback on the ACTH release [19]. The HPA axis suppression is dose-dependent and may be related to ICS potency, dose, duration of treatment, route of administration, specific drug formulation and also to any drug-drug interactions [20]. For example*,* beclometasone dipropionate induces the suppression of the HPA axis at doses > 800 μg/day in adults and > 400 μg/day in children. However, the long-term administration of ICS can induce suppression also at medium-low doses. As a consequence, ICS treatment seems to increase the risk of developing diabetes and its progression [21]. At ocular level, ICS may increase the risk of cataract incidence, although this seems uncommon in the paediatric population [22]. Psychiatric and neurological adverse events, including psychomotor hyperactivity, sleep disturbance, anxiety, aggression and depression have rarely been reported [23].

Corticosteroids also inhibit linear growth through central and peripheral mechanisms. At central level, they reduce the pituitary release of growth hormone (GH) by inducing an increase in the hypothalamic somatostatin. At peripheral level, corticosteroids down-regulate the GH receptors, inhibit the insulin-like growth factor (IGF-1) and osteoblasts activity, suppress the synthesis of collagen type I and III, and of androgens [24]. A meta-analysis confirmed that beclometasone dipropionate at a dose of 400 μg/day causes a decrease in linear growth of 1.54 cm/year compared to placebo or to non-steroidal drugs [25]. However, data available for the paediatric age show. That the slowdowns in growth rate observed in the short term may not have significant effects on the final height. Recently, a meta-analysis has highlighted a dose-dependent reduction in the rate of linear growth in preschool children treated with ICS because of mild-to-moderate persistent asthma. Linear growth decrease was reported whatever is the used ICS [26]. Nevertheless, budesonide seems to have no effects on linear growth [27] as well as fluticasone propionate [28]. In conclusion, the impact of ICS on linear growth appears to be most pronounced during the first year of treatment and less marked in the following years. Conflicting data related to the risk of ICS-induced effects on growth require to both monitor growth in treated children and use the lowest effective doses.

#### Alteration of bone metabolism

Corticosteroids reduce bone density through multiple mechanisms, including a reduced intestinal Ca^2+^ absorption, the inhibition of bone formation and an increased bone reabsorption by osteoclasts. This adverse event has been extensively investigated for systemic corticosteroids (SC), while further investigations are needed for ICS. Currently, no evidences suggest a relationship between the prolonged use of ICS and the incidence of osteoporosis.

## Indications for ICS therapy

### Wheezing

Wheezing is common in preschool children. In USA and Europe, nearly 30% of children have at least one episode of wheeze before their third birthday, and by 6 years of age the prevalence is almost 50%. In preschool children, wheezing is responsible for 50% of consultations at the paediatric office and for most of admissions to the Emergency Room and the hospital for acute symptoms. In order to reduce the disease burden and its impact on health system, research has increasingly focused on the standardization of diagnosis and treatment of preschool wheezing [29]. Unfortunately, the attempt to distinguish among several different phenotypes of wheezing (transitory; early; prolonged or persistent; atopic and nonatopic; late and intermittent) although scientifically valid, has proven to be clinically limited. Thus, an international consensus tried to simplify wheezing classification by pointing out two phenotypes on the basis of the triggering factors and timing of disease onset: Episodic Viral Wheezing (EVW) and Multiple Trigger Wheezing, (MTW) [30]. Nevertheless, few years later the same study group highlighted that the characteristics of either EVW or MTW might change over 12 months in the same subject and concluded that in most cases this classification was not helpful in addressing treatment [31]. As the phenotype may be unstable, the best approach to these patients is a continuous monitoring of the symptoms and response to the therapy.

Most international documents or guidelines, including the 2019 British Thoracic Society and Scottish Intercollegiate Guideline (BTS-SIGN) [32],the 2017 National Institute for Health and Care Excellence Guideline (NICE) [33], the 2020 Global Initiative for Asthma (GINA) report [34], the 2020 Focused Updates to the Asthma Management Guidelines 2020 (Expert Panel Working Group of the National Heart, Lung, and Blood Institute – NHLBI - administered and coordinated National Asthma Education and Prevention Program Coordinating Committee - NAEPPCC) [35], and several SR on the topic, as well as many randomized clinical trials clinical published so far, recommend treating preschool wheezing children, especially if recurrent or persistent symptoms occur, by administrating ICS that not only improve symptoms, but also ward off the onset of early pulmonary damage [36–38].

#### Are ICS the first choice maintenance treatment for wheezing in preschool children?

In order to answer the question, the consensus panel selected studies conducted on children aged less than 5 years with persistent asthma, intermittent asthma or recurrent viral wheezing. The choice of proper treatment is important in this specific age range in order:

1. To reduce hospital admissions, sleep disturbances and family concern; and
2. To avoid or reduce the airways damage, which is potentially reversible in case of timely therapeutic intervention.

The consensus panel included only studies providing strong evidences on the effectiveness of ICS compared to placebo or to other drugs, and focusing, as primary outcome, on the number of asthma exacerbations that required rescue SC. Three good methodology quality guidelines [32–35] and two SR were selected [37, 38]. GINA 2020 document was also considered being the most widespread document for the diagnosis and management of asthma in children and adolescents [34].

##### Recommendations

1. For their effectiveness and safety profile, ICS can be used for preventing recurrent preschool wheezing episodes (Very low quality of evidence, Weak positive recommendation).
2. In preschool children, ICS represent the first line drug for preventing either persistent or recurrent moderate to severe viral wheezing (High quality of evidence, Strong positive recommendation).

The 2019 BTS-SIGN reported that ICS can be used in children, also younger than 5 years old, in case of at least one of the following conditions:

- Use of inhaled β_2_ agonists 3 times per week or more;
- Cough, respiratory distress 3 times per week or more;
- Nocturnal awakenings at least once a week [32].

These recommendations are based on 10 studies and one SR of good methodology quality, that included 29 studies (with a total number of 3592 children) [36]. As alternative therapies, β_2_ agonists and anti-leukotrienes (LTRA) might be clinically beneficial also in preventing relapses in children younger than 5 years, if ICS administration is burdensome. Nevertheless, two studies included some important methodologic bias that would impose a down-grading of the level of evidence and, consequently, of the force of recommendation [39, 40].

The NICE 2017 GL suggested a two-months trial with moderate dose of ICS in children younger than 5 years old presenting with:

- Symptoms clearly requiring long-term therapy (for example, nocturnal awakenings, asthma symptoms occurring 3 times per week or more)
- Suspicion of non-controlled asthma with Short Acting β2 agonists (SABA) monotherapy (level of evidence D, opinion of experts) [33].

The GINA report 2020 recommends a low daily dose of ICS, as first choice treatment for controlling symptoms occurring in children younger than 5 years at the Step 2 (Evidence A).

The initial treatment should be given for at least 3 months to establish its effectiveness in reaching a good control. As an alternative option, the GINA report suggests to use LTRA, but in younger children with persistent symptoms, daily LTRA administration might slightly reduce the symptoms and slightly improve the need of SC compared to placebo. Indeed, in children with recurrent episodes of viral-induced wheezing neither the daily nor the intermittent use of LTRA reduce the number of exacerbations requiring oral steroids (evidence A) [41].

Different recommendations concerning starting dose of ICS are reported by the three documents. Both BTS/SIGN and GINA documents conclude that children younger than 5 years should be treated with a low daily dose of ICS (Step 2 GINA, Evidence A; BTS/SIGN, Good Practice Point). BTS/SIGN report also highlight how children with mild-moderate wheezing did not show additional clinical improvement following step up to high-dose ICS.

Conversely, the NICE GL recommends to start with a moderate dose of ICS for 8 weeks (expert opinion), in order to evaluate the stable response to ICS before shifting to the usual dose schedules.

The 2020 Asthma Guideline Update released by the US National Asthma Education and Prevention Program did not report any recommendation on the daily administration of ICS, due to lack of evidence of efficacy [35].

The SR by Kaiser et al. reported that children with persistent asthma showed a significant reduction of exacerbations with daily ICS compared to placebo- or montelukast- treated children [38]. Recently, a SR conducted on preschool children with recurrent wheezing [37] compared the effectiveness of daily ICS versus daily LTRA, for at least 3 months. Daily ICS therapy was associated with a higher number of symptom-free days, less use of salbutamol and less exacerbations compared to ICS administration or montelukast treatment (*p* = 0.001). In conclusion, the evidences confirmed that ICS are safe and effective and represent the first therapeutic choice in preschool wheezing children. The indications on the starting dose were different though low or moderate doses seemed to be both recommended for 8 weeks*,* while no proof of effectiveness or therapeutic superiority of other therapeutic options, like LTRA, was available. Only the BTS/SIGN guideline suggested LTRA administration in children younger than 5 years and /or unable to use ICS. However, the evidences supporting this recommendation are burdened by methodologic limits [33].

3. In case of less frequent but severe episodes of wheezing, even in the absence of a definitive diagnosis, a trial with daily ICS must be tried (Very low quality of evidence. Positive strong recommendation).

All documents state that all preschool children who wheeze, regardless of the definitive diagnosis, must be treated with ICS. The BTS/SIGN GL reports that most of nonatopic children aged under 5 years with recurrent episodes of virus-induced wheezing did not develop chronic asthma, therefore most of these patients would not require long term ICS treatment.

The NICE 2017 guideline recommends an 8-week trial with moderate dose of ICS to be followed by monitoring of symptoms and evaluation of the following options:

- to consider an alternative diagnosis if the symptoms do not relapse during treatment;
- to start a daily low dose of ICS as first choice if the symptoms get better but relapse within 4 weeks from the end of treatment;
- to repeat the 8-week trial with a moderate dose of ICS if the symptoms get better, but relapse after 4 weeks from the end of treatment [34].

The GINA report recommends a low daily dose of ICS, assuming that ICS, rather than LTRA, seems to be associated to a better symptom control, fewer exacerbations and less use of systemic steroids [41].

4. Children undertaking daily ICS therapy must be monitored and the drug must be used at its lowest effective dose (Very low quality of evidence. Expert opinion. Strong positive recommendation).

The BTS-SIGN 2019 GL clearly recommends to use ICS dose adequate to the severity of the presenting symptoms, starting with a very low dose and adjusting it to the lowest effective to control symptoms (expert opinion). Also the NICE guideline recommends to adjust the daily ICS dose in order to reach the lowest required dose for symptoms control.

#### Which dose, method of administration and duration of treatment with ICS?

##### Recommendations

5. Regardless of the duration of ICS treatment, a first evaluation of its effectiveness is recommended just after 4–8 weeks from the beginning of treatment, to avoid, in case of failure, the risk of uncontrolled symptoms (Very low quality of evidence. Expert opinion. Strong positive recommendation).

All guidelines agree on the importance of periodic re-evaluation of children treated with ICS, but the timing suggested for the reassessment is different and depends on specific aims. The BTS/SIGN guideline recommend to regularly reassess the patients while reducing or increasing the ICS dose in order to avoid both over- or under- treatment. It is also suggested to stop the therapy when the disease control is not achieved. Moreover, children with milder asthma and seasonal symptoms might step down the ICS dose when the allergic season is over.

The NICE guideline reported that the most appropriate time for the reassessment is 4–8 weeks after starting treatment. This recommendation is based on the clinical experience and on the Panel consensus document. However, for some patients, the reassessment could be shorter or longer compared to the suggested timing. If symptoms are controlled with the current daily therapy, any changes in dosage should be provided at least after 3 months of treatment.

The GINA report suggested to treat Step 2 and Step 3 patients initially with a daily low ICS dose and SABA add-on treatment, whenever needed (Evidence A). Patients should be treated for 3 months in order to achieve symptom control. If the initial treatment with low dose ICS for 3 months fails as exacerbations occur, suggesting incomplete disease remission, the preferred option is to double ICS dose (moderate ICS dose) and to evaluate the response after 3 months (Evidence C).

6. The *step-down* of treatment is suggested after at least 3 months of symptom control or after 3 months of absence of symptoms (Very low quality of evidence. Expert opinion. Weak positive recommendation).
7. If the patient is treated with moderate or high ICS dose, it is recommended to reduce the dose gradually and in consideration of different aspects including risk factors, compliance, access to health services, etc. (Very low quality of evidence. Expert opinion. Strong positive recommendation).
8. A 25–50% reduction of ICS dose every 3 months can be proposed (Very low quality of evidence. Expert opinion. Weak positive recommendation).

The International guidelines agree that any change in the ICS dose should be attempted at least after 3 months of treatment, but few studies evaluated the possible best way of stepping down treatment. The BTS/SIGN guideline reported one study conducted on adults treated with high ICS dose showing that in stable patients it was reasonable to try to halve the dose of ICS every three months [34]. Thus, ICS dose reduction every three months, of more or less 25–50% each time is suggested.

The NICE 2017 GL recommends to discuss with the patient (or his family or tutors) the potential risks and benefits of reducing daily therapy. Alternatively, it is recommended to consider to stop therapy with ICS only in asymptomatic patients or in subjects undertaking a daily low dose ICS monotherapy. Similarly, the GINA report concludes that even in the absence of good quality studies on the optimal duration of ICS therapy, this should last at least 3 months, or must be continued until the patient remains asymptomatic for at least 3 months.

#### What are the indications for intermittent ICS therapy?

When a preschool child suffers from asthmatic or recurrent viral wheezing, it is very hard to obtain adequate compliance on daily therapy from parents. Therefore, alternative strategies have been sought. Among these, the “intermittent therapy” consists of the administration of high doses of ICS for 8–10 days, added to SABA, as soon as the first signs of a respiratory infection occurs. Since most cases of preschool wheezing are triggered by viral infections, this approach is intended to exploit the non-genomic effect of steroids, i.e. the reduction of oedema due to induced vasoconstriction. The consensus panel searched for the most relevant studies evaluating the effectiveness of the pre-emptive therapy with ICS high dose for 7–10 days at the first signs of respiratory infections rather than daily use of low dose ICS (as intermittent use) or than placebo. Clinically relevant outcomes were considered, i.e. reduction of frequency, severity, duration of exacerbations and safety data on linear growth rate.

The NICE 2017 GL, the GINA 2020 document [33, 34] and four SR, including three of moderate quality [38, 42, 43] and one of high methodologic quality were considered [44]. The BTS/SIGN GL was excluded because the absence of specific recommendations.

Analysed studies were heterogeneous and not comparable because of different steroid molecules, routes of administration, doses and follow-up periods reported [32].

In children aged 0 to 4 years with recurrent wheezing triggered by respiratory tract infections and no wheezing between episodes, the 2020 US Update conditionally recommends starting a short course of daily ICS at the onset of a respiratory infection with as-needed SABA for quick-relief rather than only as-needed SABA (Recommendation 9. Conditional recommendation. High certainty of evidence: three RCTs) [35].

##### Recommendations

9. In preschool children with intermittent wheezing or recurrent moderate-severe virus-induced wheezing, intermittent therapy with high dose ICS could be used for 7–10 days as soon as first signs of respiratory infection occur (Moderate quality of evidences. Weak positive recommendation).

Evidences of moderate/low quality suggest that also in preschool children with intermittent or virus-induced recurrent - but not persistent – wheezing, high dose ICS given at the beginning of a respiratory infection for 7–10 days could be beneficial. ICS reduced severity and frequency of exacerbations which required oral steroids, but did not influence the rate or the risk of exacerbations and the use of rescue therapies.

The NICE 2017 GL addressed the question whether in preschool children with asthma or wheezing the use of intermittent rather than ICS daily therapy might be more effective and less expensive. The Panel stated that proofs were insufficient to confirm that the intermittent use is better, worse or equivalent to daily administration of ICS.

The GINA 2020reported that in subjects at Step 1 (with intermittent wheezing and no intercritical symptoms) in whom SABA is unable to control symptoms, ICS intermittent therapy can be considered. However, for children in the step 2 (uncontrolled symptoms), the document recommends, as first choice treatment, the regular administration of daily low dose of ICS, plus SABA as needed (Evidence A). In conclusion, low dose ICS should always be considered the first option treatment, while intermittent therapy at high dose might be an alternative option in case of intermittent wheezing, taking into account that the risk of exacerbations is similar to the daily low dose (Evidence A).

In agreement with these data, Rodrigo et al. reported in their SR a comparable rate of exacerbations among patients receiving either daily low dose or intermittent high dose ICS [42]. Similarly, the Cochrane SR by Chong did not find significant difference in the hospital admissions or in the quality of life reported by the parents [43]. However, in preschool children with frequent wheezing episodes, the intermittent use of ICS at the first symptoms halved the need of oral corticosteroids and was associated with an improvement of daily and nocturnal symptoms [35]. Kaiser et al. evaluated 22 studies on children with asthma aged under 6 years or with recurrent viral wheezing older than 2 years in order to rule out bronchiolitis [38]. The subgroup analysis on children with moderate to severe intermittent or virus-induced wheezing treated with beclomethasone or budesonide (high-dose daily or intermittent), showed reduced exacerbations compared to placebo. The most recent SR of the Agency for Healthcare Research and Quality evaluated the effectiveness of intermittent therapy with ICS in different populations (aged from 0 to 4 years with recurrent wheezing and older than 5 years with persistent asthma) [36]. Compared to SABA therapy as needed, adding intermittent ICS reduced the risk of exacerbations and the need of oral steroids while improving the quality of life of children younger than 5 years with recurrent wheezing during a respiratory infection. In comparison to daily ICS therapy, the intermittent ICS use did not show significant differences in:

- risk of exacerbation needing oral steroids (low quality evidences) or nocturnal therapy with SABA (low quality evidences);
- hospital admissions (low quality evidences);
- use of *rescue* daily therapy and nocturnal therapy (low quality evidences).

Authors concluded that also in preschool children with intermittent or virus-induced recurrent wheezing, but not persistent symptoms, high dose ICS administered at the beginning of a respiratory infection and prolonged for 7–10 days reduces the risk of exacerbations requiring oral steroids, but not the total rate and risk of exacerbations and the use of *rescue* drugs.

10. Due to the absence of data on long term safety of ICS in preschool children with highly recurrent episodes of virus-induced wheezing, intermittent therapy with high dose ICS must be stopped if the episodes do not decrease in frequency (< 1–2/month) (Very low quality of evidences. Expert opinion. Strong negative recommendation).
11. Preschool children undertaking intermittent high dose ICS therapy must be always carefully monitored (Very low quality of evidences. Expert opinion. Strong positive recommendation).

Data on the safety of high dose ICS intermittent therapy are uncertain as few studies reported a reduction of the linear growth rate [45]. Concerning adverse events, three studies cited in the SR [46–48] did not report significant differences in linear growth after 1-year of therapy versus placebo or versus daily low dose ICS. Because intermittent therapy requires doses 4–8 times higher than daily low dose, high frequency of respiratory infections and/or of wheezing episodes could result in a higher total intake of ICS, reducing the benefit-risk ratio of this preventive intervention.

Moreover, differently from what happens in asthmatic school children, the administration of ICS to children with virus induced moderate to severe wheezing is necessarily related to the infective episodes (that are not influenced by ICS) and not to asthma, so the direct relationship between intervention and outcome decades (administration also without wheezing). Since preschool children suffer from very frequent infections of the upper respiratory tract, it is highly probable that ICS courses are so many during a year that might significantly increase the risk of adverse events. Therefore, recommending the extensive use of this approach is burdensome.

### Bronchial asthma

Bronchial asthma is a chronic inflammatory disorder of the airways. In Italy, the prevalence of paediatric asthma ranges from 9 to 10% [49]. The rate of hospital admission for asthma is still high with an estimated cost of 1–2% of the total social-health cost, including parents’ absence from work [41, 50, 51].

Therefore, the need to strengthen the approach to asthma is recognized by the scientific community and many guidelines or official documents have been published so far on the appropriate drugs, dosages and devices for this disease.

The control of asthma is an emerging problem worldwide A recent survey reports that 47% of US children aged 0 to 4 years, 38% of those aged 5 to 11 and 35% of those aged 12 to 17 have uncontrolled asthma [52]. Likewise, many Italian studies showed similar results [53, 54]. Many factors contribute to poor disease control, such as overestimation of control, poor evaluation of symptom severity, poor adherence to therapy, often as a result of unfounded concerns about ICS toxicity [55]. However, while high ICS dosages might be of some concern, it is known that when ICS are prescribed at the minimal effective dose and duration, their benefits overcome the potential side effects [56, 57]. Indeed, repeated exacerbations and persistence of airway inflammation can induce a progressive reduction of respiratory function that over time becomes irreversible [58]. Furthermore, inadequate asthma control significantly impacts on public health costs [56, 58].The most recent GINA document [34] highlights the necessity to improve the adherence to therapy and urge the healthcare givers to empower an efficient communication with patient and his parents, in order to improve the *self-management* of the disease Clinicians must undertake “personalized” medicine, based on the single child needs: therapy should be modulated on severity of the disease and using *step up* or *step down* strategy according to the clinical response [34].The continuous reassessment, involving child and his *caregivers*, is crucial. The GINA report exemplifies reassessment concept as “circular” route of assessment organized on three crucial points: - evaluate the patient, − personalize the treatment, − reassess the treatment response. Follow up data must be shared between Hospital and general paediatricians and a suitable network for childhood asthma on national territory should guarantee clinical assistance while limiting admission to hospital and reducing the costs of public health.

#### Is it appropriate to use ICS for acute asthma treatment in patients with intermittent asthma?

**Is it appropriate to step up ICS dose in acute asthma treatment of patients with persistent asthma already undertaking d**ai**ly therapy?**

Although national and international guidelines report therapy choice recommendations based on the severity of asthma, in the clinical practice the association of SABA and ICS is often prescribed regardless of the severity of the acute attack. Therefore, in order to answer the first question, the consensus panel evaluated the proves of effectiveness and safety of SABA + ICS association, compared to standard therapy of asthma attack (SABA alone), with or without bromide ipratropium and/or SC. The following outcomes were considered: clinical effects, use of systemic corticosteroid, hospital admissions and permanence in Accident and Emergency (A&E) unit. As for the second question, studies on the effectiveness of the up-dose of ICS in patients already under daily ICS treatment for persistent asthma were searched. Finally, the following documents were accepted and considered: SIGN-BTS [32] and SIP 2016 guidelines [59] both of good methodologic quality; Technical Report GINA 2020 [34]; NIH 2020 [35], 2 RS [60, 61] and 6 RCT [62–67].

Scientific evidences on these topics are very heterogeneous for all PICO elements: 1. Population: age (adults/children); asthma step (step 2/not specified); severity of asthma attack (mild, moderate, severe); 2. Intervention: ICS molecule (budesonide, fluticasone, etc.), dose (low/ increased), association [SABA + ICS, SABA + ICS + Ipratropium/oral corticosteroids (OCS, not specified)]; 3. Comparison: SABA + placebo, SABA + SCS, SCS alone, not specified; 4. Outcomes: hospital admissions, FEV1, severe adverse events, SCS dosage, increasing dose of SCS, SABA dosage, duration of the attack/hospital admission, specialist examination, quality of life.

Because of this wide heterogeneity, the limited number of studies in paediatric age and the difficulty to delineate the clinical practice, it was rarely possible to formulate strong recommendations.

##### Recommendations

12. Inhaled corticosteroids (ICS) should not be administered alternatively or added to SC during moderate-severe acute asthma attack (Low quality of evidence. Weak negative recommendation)
13. In children undertaking daily ICS treatment, it is not necessary to increase the dose during acute attack (Moderate quality of evidence. Weak negative recommendation).

SIGN-BTS 2019 and SIP 2016 guidelines (GL), both of good methodologic quality, did not recommend ICS use during acute asthma attack. BTS do not recommend the use of ICS as alternative or additional treatment to OCS, too. SIP GL discourage the use of ICS for the acute attack of asthma in children older than 2 years. Moreover, it is not recommended to increase the dose in patients undertaking ICS, while it has been suggested to take the usual dose of ICS (Recommendation number 9. Weak Negative). The quality of evidence is weak because of the heterogeneity of the interventions [59].

Based on two recent studies [68, 69], the GINA 2020 report added a new recommendation for patients older than 12 years with asthma exacerbations, encouraging for this population the use of budesonide-formoterol at each acute episode and overcoming the old recommendation of using SABA monotherapy. On the contrary, for children aged 6–11 years, to add low dose ICS at step 1 was indicated as optional treatment. It is mandatory to underline that the above mentioned studies are limited by important bias: they have been conducted on adolescent and adult patients at step 2. So, in the absence of further evidences, there is no agreement on the transferability of the results also on paediatric patients at step 1. In conclusion, GINA 2020 report recommended the association budesonide-formoterol at any asthma episode, even mild, essentially as an alternative to daily therapy. Moreover, the use of ICS was considered an “available option” also for asthma exacerbations in children 5 years and younger if they did not respond to SABA monotherapy.

Two SR evaluated the effectiveness of ICS in acute asthma. Su et al. compared hospital admission rate of children treated with high dose ICS (400–2000 μg) to that of children treated with placebo or OCS (prednisone 1–2 mg/kg/die) and found that the hospital admission rate was not significantly different between children treated with ICS compared to children OCS treated, but was lower in ICS treated patients- compared to placebo- treated children [60]. Kew et al. studied patients with persistent asthma undertaking ICS daily therapy [61]. At the first signs of exacerbation, intervention group received double ICS dose, whether control group continued daily regular dose. The outcome was the reduction of the severity of the exacerbations and/or the use of OCS. As for the need of OCS, no statistically significant difference was found between the two groups (OR = 0.89, 95% CI = 0.68–1.18). To date, evidences support the use of ICS, only when comparing the association SABA + ICS to SABA + placebo. Patients that could take advantage from ICS administration in acute attack were the ones not treated with ICS as daily therapy or those that did not take OCS as acute attack therapy.

14. In children who do not take ICS as *controller* (Step 1), ICS can be added instead of SC in case of mild or moderate acute asthma attack that do not improve within the first hour with SABA monotherapy (Moderate quality of evidence. Weak positive recommendation). In case of failure (worsening of asthma symptoms), SC will be promptly administered (Experts opinion. Strong positive recommendation).

Concerning the management of worsening asthma and exacerbations in acute care settings, GINA report stated that the optimal treatment consists in repeated administration of SABA and eventually OCS. ICS are not recommended. However, in emergency settings, administration of high dose ICS within one hour from symptoms onset reduced hospital admission (Evidence A). If ICS were added to OCS, the clinical results were contrasting (Evidence B). In conclusion, as for appropriateness of ICS in acute asthma, GINA suggested the use of ICS only in Emergency Departments. Even though ICS were well tolerated, the cost did not justify the treatment. A specific cost-benefit analysis was not available, but it was estimated that ICS + SABA association increased direct costs of inhalant therapy of the 550% and that ICS cost was the 1.000% of OCS. In conclusion, the algorithm of the management of the exacerbations in the emergency setting did not foresees ICS administration, while including SC.

Finally, 6 RCTs [62–69] evaluated ICS addition (mostly budesonide) to standard therapy at different doses and with different ways of administration in acute asthma attack compared to standard therapy plus placebo or saline solution in patients of different age (7–72 months, 2–18 years and 5–18 years) admitted to ER. The results obtained, concerning time spent in ER, rate of hospital admission, respiratory function tests, were not in agreement.

Despite the heterogeneity of these studies, the consensus panel identified three clinical subgroups of patients that reflect the most commonly encountered situations in clinical practice: 1. untreated patients with a mild asthma episode, to whom salbutamol spray is not prescribed, except in case of worsening; 2. untreated patients with a moderate asthma episode, to whom salbutamol spray and OCS are generally prescribed; 3. patient ongoing daily therapy presenting with an asthma episode to whom salbutamol spray added to ICS, administered as controller, are prescribed. In the group 2, taking OCS or ICS at low doses did not show significant clinical differences. Furthermore, what molecule, dose, duration of treatment with ICS in the management of asthma in ER are still to be defined [70, 71].

#### In asthma uncontrolled with standard doses of ICS, is it preferable to double ICS dose or to add a different molecule?

Possible therapeutic options in paediatric patients with uncontrolled asthma have been consistently debated leading to many and different recommendations from the guidelines published so far.

The consensus panel selected studies conducted on children older than 5 years or on teenagers, undertaking ICS therapy and with poor clinical control. Scientific evidences were searched on the effectiveness and safety of the add on therapy -from step 2 to step 3- for persistent asthma with *Long Acting B2-Agonists* (LABA) or anti leukotrienes (LTRA) plus low ICS doses compared to moderate ICS doses or high doses. Considered clinical outcomes were: number of asthma exacerbations needing β2-agonists or systemic steroid as *rescue-therapy*; number of hospital admission including ER admission. Due to their optimal methodologic quality, only two guidelines have been included in the final analysis, namely the BTS-SIGN [32] and the NICE [33]. In addition, also the GINA Technical Report 2020 [34] recommendations have been considered. Furthermore, we included two SR [72, 73] and two out of the 58 RCTs found on this topic [74, 75]. In the last two years no trials have been published focusing on LABA or LTRA effectiveness as adding therapies from Step 2 to Step 3 treatment of Asthma.

##### Recommendations

15. In children older than 5 years, with not controlled asthma despite low dose ICS treatment, it is possible to add LABA or LTRA (High quality of evidence. Weak positive recommendation). It is possible to double ICS dose in case of failure of add-on therapy with LABA or LTRA (Moderate quality of evidence. Weak positive recommendation).
16. In children with persistent asthma not controlled by ICS low dose treatment, high dose ICS should not be used (Moderate quality of evidence. Strong negative recommendation).

In order to achieve a stable asthma control, BTS-SIGN GL suggested (weak recommendation; grade B) to optionally use LRTA or LABA and to increase the dose of ICS only if the abovementioned add-on therapy fails. Consensus Panel recommend that LABA should be used only in addition to ICS. In children, the options for an *add-on* therapy were limited to LABA and LTRA, with evidences supporting both options, but not sufficient to affirm the preferential use of one treatment rather than another (LoE for 5–12 years old = 1++). The SR Cochrane by Chauhan et al. evaluated the effectiveness of LABA versus LTRA add-on therapy. The authors concluded that, in adults, adding LABA is better than LTRA in reducing exacerbations treated with OCS [76]. Other studies observed that LABA were also able to improve the quality of life, but this outcome was statistically significant only in one study [33]. Because of the limited number of paediatric trials, it was not possible to suggest the best add-on therapy in children.

Evidences for population aged 5–16 years were few. The Consensus panel include only one double blind “MASCOT study” [77], with low number of subjects enrolled (around 50) and even lower number of available results for each outcome*.* A group of school age children was treated with ICS at low dose, another group with ICS + LABA and finally a group with ICS + LTRA, for 48 weeks. For none of the *5 outcomes analyzed* (severe exacerbations treated with OCS, quality of life, hospital admissions, variations of the FEV_1_ and infections) the results were significant, and only a trend supporting LRTA administration was observed. The authors concluded that ICS low doses and ICS + LTRA association lead to a better clinical outcome compared to the ICS + LABA association, particularly in severe exacerbations, quality of life and hospital admissions. Nevertheless, the quality of evidence was very low. Based on this, the NICE GL supported the preferential use of LTRA in the paediatric age and recommended to add LTRA at Step-up also because of a more favorable cost-effectiveness ratio. Nevertheless, an economic analysis restricted to paediatric age was not available due to the paucity of paediatric data. Furthermore, the weak recommendation to start the therapy with LTRA was immediately followed by another weak recommendation of shifting to LABA in case of LTRA failure after a 4–8 weeks monitoring observation.

GINA Technical Report 2020, instead, contemplated the increase of the daily dose of ICS following the *add-on therapy* with LABA.

For the option to increase ICS dose, Technical Report GINA 2020 suggested, without expressing a clear recommendation, to increase ICS dose as an alternative option to ICS + LABA at Step 3, while BTS-SIGN GL recommends to increase ICS only when the association LABA+ICS fails. The NICE GL expressed negatively about increasing ICS dose, without giving a specific recommendation. This negative opinion was based on a single work [78] which compared two groups of children with poorly controlled asthma treated with different doses of ICS. Statistically significant differences were observed but clinically irrelevant (use of *reliever* medications, mild improvement of FEV1 and PEF, number of infections).

In NIH guidelines, the recommendation 12supports the administration of ICS-formoterol in a single inhaler, *“single m*ai*ntenance and reliever therapy (SMART)”,* in children aged 4 years and older with moderate to severe persistent asthma. This treatment, as both daily controller and reliever therapy, was compared to either

1. a higher-dose ICS as daily controller therapy and SABA for quick-relief therapy
2. the same-dose ICS-LABA as daily controller therapy and SABA for quick-relief therapy

(Strong recommendation, high certainty of evidence for ages 12 years and above, moderate certainty of evidence for ages 4 to 11 years).

In conclusion, SMART is appropriate for Step 3 (low-dose ICS-formoterol) and Step 4 (medium-dose ICS-formoterol) treatment, and might not be necessary for subjects whose asthma is well controlled on conventional maintenance ICS-LABA with SABA as quick-relief therapy.

Among SR, only two were included [72, 73]. The first one compared the *add-*on strategy with LTRA to increased ICS dose and concluded in no difference between the two interventions for any studied outcome. The second one, of better quality, gathered 16 RCTs for a total of more than 22.000 patients older than 4 years, in which a comparison among *M*ai*ntenance and Reliever Therapy* (MRT with ICS + LABA for limited periods of time) versus continuative ICS alone (at the same dose and at increased dose compared to MRT) versus continuative ICS + LABA (using ICS at the same dose and at increased dose compared to MRT) was made. In the metanalysis the effectiveness of MRT compared to increased ICS dose was analyzed highlighting that in patients older than 12 years and adults, MRT groups had 40% less risk to develop severe asthmatic exacerbations. Also in patients aged 4–11 years MRT better reduced severe asthmatic exacerbations compared to ICS alone (results from a single study).

Sobieraj and colleagues concluded that MRT is associated to a lower risk of asthmatic exacerbations and that the evidence for patients aged 4–11 years old are limited [73].

Beyond the two primary studies specifically related to the question to be addressed, one study [74] reported evidences from 3 previous RCTs [79–81] that had enrolled patients already undertaking ICS therapy and presenting with poor symptomatic control. This *post-hoc* analysis compared ICS (moderate dose) + LABA association (MRT) to high dose ICS on 1239 patients aged 4 to 80 years (with results unfortunately not separated for age). The overall methodologic quality of the work was low. The main result was a statistically significant difference in favor to the MRT in reducing severe exacerbations in a subgroup of patients that, in *run-in* phase, had used *reliever* therapy for more than once a day.

The second study [75], of moderate methodologic quality, was part of a project named STICS (STep-up Yellow Zone Inhaled Corticosteroids to Prevent Exacerbations, in the Asthma Net Steering Committee del National Heart, Lung, and Blood Institute). Authors studied the effectiveness of different management strategies in children aged 4 to 11 years with persistent asthma (fluticasone propionate at a dose of 44 μg per inhalation, two inhalations twice daily - low-dose group - or use a quintupled dose - high dose group - fluticasone at a dose of 220 μg per inhalation, two inhalations twice daily) for 7 days at the early signs of loss of asthma control). Non-significant differences were found for the main clinical outcomes.

### Chronic rhinitis

Rhinitis is characterized by rhinorrhoea, nasal itching, sneezing and nasal obstruction. Different phenotypes are well defined: allergic rhinitis (AR), “non-allergic rhinitis” (NAR), Local Allergic Rhinitis: (LAR), a subtype of allergic rhinitis with only local symptoms in the absence of systemic atopic sensitization. According to the ARIA Guidelines’ classification, rhinitis is defined persistent when symptoms last longer than 4 days per weeks and recur for more than 4 weeks [82]. In Europe, 80 million people suffer from allergic diseases and 75% of them are affected by AR and also an Italian epidemiological study (SIDRIA 2006 reported a high percentage of children older than 6 years old affected by AR. This percentage increased at the age of 13–14 years. Allergic rhinitis (AR) is characterized by inflammation of the nasal and contiguous mucosa (rhinosinusitis); consequently, the risk of conjunctivitis and asthma is increased. The therapy includes nasal steroids, possibly associated with topical or systemic antihistamines or topical anticholinergics.

#### For which rhinitis phenotype are nasal steroid indicated?

To answer the question, the consensus panel included 5 Guidelines. The reference population of the Guidelines consist of patients with seasonal (Seasonal Allergic Rhinitis - SAR) and persistent allergic rhinitis (Persistent Allergic Rhinitis PAR), without age differentiation [82–86]. Only the BSACI 2017 reported specific recommendations for children and for non-allergic rhinitis (Non-Allergic Rhinitis -NAR) [84].

##### Recommendations

17. For the level of efficacy and the safety profile, ICSs monotherapy is indicated as 1st choice in seasonal and perennial allergic rhinitis, preferably to the use of nasal antihistamines (Low quality of evidence. Low positive recommendation), to oral antihistamines and to LTRA (Moderate quality of evidence. Strong positive recommendation).
18. ICS monotherapy is indicated as 1st choice also in persistent non-allergic rhinitis, preferably to nasal antihistamines (Low quality of evidence. Weak positive recommendation), oral antihistamines and nasal decongestants that have been proven ineffective. (Moderate quality of evidence. Strong positive recommendation).

All GLs agreed in indicating monotherapy with ICSs as first choice in SARs and PARs (strong recommendations). Continuous use is more effective than intermittent one.

BSACI 2017 states that topical steroids are effective also in patients with NAR (level 1b). In particular, the authors speculate that, despite negative prick tests, subjects presenting with an underlying eosinophilic inflammation, respond better than subjects with low levels of nasal eosinophils, as also reported by Tantilipikorn et al. [87]. Rivera Ramirez confirms that ICSs in adults and adolescents presenting with PAR are more effective than placebo in controlling symptoms (*p* < 0.001) both in the short (less than 6 weeks) and in the long term observation, (52 weeks or more) even if the improvement is clinically inconsistent [88].

Finally, Khattiyawittayakun et al. in a meta-analysis including 5 paediatric studies (1868 patients), confirm that the effectiveness of the ICSs is dose dependent, therefore increasing if doubling the dose [89].

As an alternative to ICSs, GL ICARs report that intranasal antihistamines can be used in the treatment of AR (Recommendation 1A- 1-B) [85]. The document suggests also the use of intranasal decongestants (IntraNasal Decongestant, IND) as a short-term treatment option and being aware that prolonged therapy may lead to iatrogenic Rhinitis (Option B). Juel-Berg et al. evaluated 2 studies (307 patients with AR) in order to analyse the effectiveness of ICSs compared to oral antihistamines and confirmed the superiority of steroids in controlling nasal symptoms [90].

As for the possibility of adding other drugs, only the ARIA GL pointed out that the association of intranasal corticosteroid with antihistamine H1is preferable to intranasal antihistamine H1 alone in patients with SAR [83]. The safety profile was good, as no serious adverse events were reported apart from occasional nosebleeds [91].

#### Is it recommended to add other drugs to inhaled corticosteroids?

To answer the questions, the consensus panel looked for data regarding all possible treatment associations: 1-Intranasal ICS-IntraNasal AntiHistamine (INAH) association versus ICS in monotherapy; 2- ICS- Oral Antihistamine (OAH)association versus ICS in monotherapy; 3- ICS - Nasal decongestants association versus ICS in monotherapy; 4- ICS Association - Topical Anticholinergic (Ipratropium Bromide, IPB) versus ICS in monotherapy. Five GL [82–86], 2 SR [92, 93] and 2 primary studies were selected [94, 95].

##### Recommendations

19. The intranasal ICS-INAH association could be used in patients suffering from persistent allergic rhinitis, whose symptoms are not controlled by antihistamine or ICS monotherapy (moderate quality of evidence. Weak positive recommendation)

The GL recommended that the ICS-INAH combination should be used in patients presenting with SAR or PAR whose symptoms are not controlled by INAH or ICS monotherapy. Also the RS by Seresirikachorn et al. [92] confirmed the greater effectiveness of the intranasal INAH-ICS association compared to the ICS monotherapy, even though the clinical relevance, measured as quality of life (QoL) and nasal flow, seemed to be modest. Two recent primary studies on children presenting with RA confirmed the superiority, even if at the limit of significance, of the azelastine-fluticasone association compared to the steroid alone in the control of symptoms [96, 97]. As for patients with NAR, only the BSACI 2017 reported that one-year therapy with Fluticasone/Azelastine association can reduce symptoms [84].

20. The ICS-OAH combination should not be used in patients with persistent allergic rhinitis, in which the symptoms are not controlled with oral antihistamine or with ICS monotherapy (low quality of evidence. Weak negative recommendation)

The recommendations of the guidelines are argumentative. In general, the ICS-OAH association is not recommended. The ICS-OAH association compared to ICSs in monotherapy did not increase clinical outcome when analysing subgroups of patients presenting with RS. However, the ARIA GL conditional recommendation (with very low quality of evidence) in SAR or in persistent moderate/severe rhinitis not controlled by topical intranasal corticosteroids or associated with ocular symptoms [83]. OAHs seemed to be ineffective in NAR [84].

21. The association ICS - topical anticholinergic can be indicated in patients suffering from allergic rhinitis with persistent aqueous rhinorrhea (very low quality of evidence. Weak positive recommendation)

IPB is licensed for children older than 12 years. The regular intranasal use can be effective in decreasing rhinorrhea but has no effect on other nasal symptoms. Therefore, IPB can be indicated as an “add-on” therapy for allergic rhinitis when aqueous rhinorrhea persists despite topical steroids and antihistamines are correctly administered [84, 85].

22. The association ICS-nasal decongestants should not be used in patients with allergic and non-allergic rhinitis with persistent nasal obstruction (low quality of evidence. Weak negative recommendation)

Topical formulations of IND relief nasal congestion through vasoconstriction in a few minutes, faster than intranasal steroids. The 2017 BSACI GL states that IND may be indicated in case of Eustachian tube dysfunction (level of evidence D), to increase nasal patency before irrigation (Grade D) or before administration of nasal steroids; these recommendations are based on expert opinion. These drugs are indicated for maximum 10 days and are ineffective in NAR [84]. The most recent meta-analysis by Khattiyawittayakun et al. showed no benefits compared to ICS monotherapy, neither in NAR nor in PAR [93].

### RHINOSINUSITIS

The anatomical-functional affinity between the nose and paranasal sinuses is the reason why rhinitis is almost always associated with sinusitis. Viral infections of the upper airways are the most common trigger of acute rhinosinusitis. If the symptoms last more than 10 days, mucus becomes dense, greenish-yellow or frankly purulent and drips into the nasopharynx (post-nasal drip), thus being responsible for persistent cough, halitosis and sometimes headache. Diagnosis is clinical and computed tomography scan (axial and coronal projections) is the gold standard in serious cases. Antibiotics are the pivotal treatment and should be administered up to 4–6 weeks in chronic disease. Some recent clinical studies highlighted the advantages of the topical antibiotic-steroid association not only in rhinosinusitis associated with allergic rhinitis, but also in post-infectious symptoms of non-atopic subjects. Adding steroids in rhinosinusitis reduces the mucosal inflammation.

#### Is nasal steroid indicated in acute rhinosinusitis?

The main problem in selecting evidence in order to formulate recommendations has been the lack of homogeneity for the clinical definition of rhinosinusitis. Furthermore, only few studies on paediatric and adolescent patients up to 21 years of age have evaluated the efficacy of therapy with ICS, as exclusive or additional topical treatment, in terms of clinically relevant outcomes. To answer the question, the consensus panel selected the following papers: one Consensus document [the International Forum on Allergy and Rhinology: Rhinosinusitis (ICAR: RS)] [94], one SR [95] and 2 studies published when the bibliographic searches of the two above mentioned documents were concluded [98, 99].

##### Recommendations

23. ICS should be indicated in the therapy of acute rhinosinusitis alone or in addition to systemic antibiotic therapy (low quality of evidence. Positive recommendation weak)

Although ICAR document includes a chapter for paediatric age, it does not provide recommendations, but only a general indication for the use of ICS in acute rhinosinusitis [98].

The Cochrane SR [95], of moderate quality, included 4 studies (1943 patients) assessing the efficacy of ICS, compared to placebo or no intervention, administered for 15 or 21 days in subjects with a clinical, or CT confirmed, diagnosis. The meta-analysis demonstrated an improvement in symptoms in patients treated with ICS compared to those treated with placebo. However, the number of children studied was very low.

Primary studies, both conducted on paediatric patients, had critical issues [98, 99]. Rahamati et al. randomized 100 children (2–14 years old) with RSA, in 2 treatment groups: amoxicillin with fluticasone nasal spray twice daily for 14 days (intervention) versus amoxicillin alone for 14 days (controls) [98]. The symptom score improved significantly in the intervention group compared to the controls. However, it is not mentioned if the study has been conducted as blind trial. Tugrul et al. randomized 104 children in 2 treatment groups: antibiotic plus decongestionants versus fluticasone plus saline irrigation [99]. The difference in clinical scores was significant only at 7 days and for some symptoms. However, clinical improvement was not quantified and was not confirmed during the follow up [99]. In conclusion, evidences suggested that in paediatric patients ICS can be indicated in the acute rhinosinusitis, but their effectiveness seems generally modest. In some studies, ICS were combined with 0.9% NaCl solution nasal irrigation, but additional RCTs are needed to confirm the finding [100].

### Is nasal steroid indicated in chronic rhinosinusitis?

To address this question, one Consensus document [the International Forum on Allergy and Rhinology: Rhinosinusitis (ICAR: RS)] [94] and 2 Systematic Review (RS) of moderate quality [101, 102] were selected.

#### Recommendations

24. ICS can be indicated in the treatment of chronic rhinosinusitis in children and adolescents, especially in patients without nasal polyps (CRsP) and /or undergoing surgery. There is not enough evidence to suggest that the different types of corticosteroid molecules or spray vs. aerosols have different effects. The lower doses have similar efficacy and fewer side effects (very low quality of evidence. Positive recommendation weak)

The SR and RCTs available have severe limitations in addressing the question concerning ICS efficacy. First of all, available recommendations derive from studies conducted on adults and the transferability of the findings to a paediatric population cannot be taken for granted. Furthermore, methods of administration, duration of therapy and of follow up differ consistently, especially in consideration of the possible suppression effects on the hypothalamic-pituitary axis during paediatric age. For the management of chronic rhinosinusitis with nasal polyposis (CRsP), the ICAR document highlighted how direct sinus administration of ICS (drops or sprays) showed conflicting outcomes in different studies. The document concludes that ICS may be an option in more complex cases of CRsP or after failure of treatment with other drugs (Aggregate grade of evidence: A. Level 1a: 2 studies, Level 1b: 2 studies) [94]. However, the nasal irrigation seemed to be a better option in postoperative patients (grade of evidence C), as the studies evaluating ICS for irrigation showed in these patients a significant improvement in QoL, subjective symptom scores (symptom scores = 2.5 ± 1.1 vs 1.4 ± 1.0) and endoscopic findings [102]. Common side effects were nosebleeds and headaches for both methods of administration, but irrigations have a higher cost than sprays. In Chronic Rhinosinusitis without Nasal Polyps (CRSwNP), the consensus recommended ICS before or after sinus surgery (aggregate degree of evidence: A (level 1b: 36 studies, level 2b: 4 studies). The standard administration (drops and spray) of ICS improved the symptom score, endoscopic findings, QoL, as well as olfactory tests. The therapy reduced relapses of respiratory symptoms and polyps formation. Concerning topical administration method, nasal aerosols and dry powder inhalers seemed to be more effective than nasal sprays in controlling symptoms [94]. Mometasone, fluticasone and ciclesonide have shown efficacy comparable to that of older molecules (budesonide, beclomethasone, betamethasone, triamcinolone and dexamethasone) [102].

As for non-standard treatment modality (irrigation and nebulizers), the consensus cited only one level B study with inconclusive results [94]. Irrigation is an off label method of ICS administration and represents a possible therapeutic option only after sinus surgery. Aggregate degree of evidence: B (Level 1b: 1 study, Level 4: 5 studies). Systemic CS treatment was also studied with variable results and hypothetical risks of systemic adverse events ranging from < 1% to up to 40 to 50%. However, nasal biopsy following long-term ICS administration did not show iatrogenic damage to the nasal mucosa [101]. These data were confirmed by the Chong review on 13 studies (2508 participants) that only reported a relative risk of nosebleeds equal to 2.74, 95% CI 1.88–4.00.s (high quality evidence) [102]. The review included only 1 paediatric study (6–17 years) addressing ICS safety [103]. Authors confirmed the increased risk of nosebleeds -without any other side effects- in children treated with mometasone furoate compared to placebo.

### Adenoid hypertrophy

Adenoid hypertrophy (AH) is one of the most frequent cause of upper airways obstruction in childhood, and it is characterized by oral breathing, rhinolalia, hearing loss and sleep disorders (snoring; obstructive sleep apnea syndrome, OSAS). Sleep disorders may worsen the quality of life as well as neuro-cognitive development and growth. AH can also cause obstruction of the Eustachian tube, eventually resulting in otitis media with effusion (OME) and transmissive hearing loss. Children should be tested for AH in case of persistent nasal obstruction (> 3 months): rhinoscopy with rigid or flexible endoscopes is the gold standard testing, while lateral X-ray of the neck is considered useless and harmful. Nasal obstruction associated with AH can be assessed by the nasal obstruction index (NOI), a four-point scale (1no-, 2 mild-, 3 moderate-, 4 severe –obstruction) which corresponds to the grading of the adenoid hypertrophy occupying the choanal area (< 25% = 1; < 50% = 2; < 75% = 3; > 75 = 4). Therapy depends on the degree of hypertrophy/obstruction being medical at grades 1–3 and surgical at grade 4.

#### Is topical treatment with ICS indicated in adenoid hypertrophy?

In order to address the question, the consensus panel analyzed studies on children and adolescents with moderate-severe AH symptoms diagnosed with X-ray and/or rhino-pharyngeal endoscopy, and/or with an indication for adenoidectomy. The GL including other diseases such as OME and OSAS published by 2014 were also analyzed. One GL [104] and 4 Systematic Reviews, 3 of them of moderate quality [105–107] and 1 of low quality [108] have been selected. Any other relevant RCT not included in the SR was also included. The panel considered as critical outcomes: reduction in the number of adenoidectomies; improvement of the sleep apnea/hypopnea index (AHI) confirmed by polysomnography; reduction of the NOI index and –very important- of AH grading; adverse events, improvement of nasal symptoms; resolution of the endotympanic effusion confirmed by tympanometry.

##### Recommendations

19. ICS can be administered in children, especially in school age, with moderate/ severe AH in order to:
  - treat mild / moderate obstructive sleep apnea

(Very low quality evidence. Weak positive recommendation)

b. reduce adenoid hypertrophy

(Low quality evidence. Weak positive recommendation)

iii. improve nasal obstruction symptoms

(High quality evidence. Strong positive recommendation).

AHI reduction in children with OSAS and AH has been evaluated in 2 systematic reviews and 2 studies.

Two SR of moderate quality [105, 106] evaluated the effectiveness of ICS, compared to placebo by considering signs/symptoms: size of the adenoids and nasal obstruction symptoms AHI, reduction of OME and improvement in quality of life. For every sign /symptom, evidences were quite limited and results were inconclusive. Despite current evidences support the therapeutic intervention, evidences on long-term efficacy and safety are lacking. Two additional trials, published after the SR [109, 110], confirmed the efficacy of mometasone furoate (MF) spray, 50 μg, 2 sprays per nostril in the evening, respectively for 4 and 3 months. Authors observed a statistically significant reduction in AHI compared to placebo: respectively 2.7 ± 0.2 to 1.7 ± 0.3: *p* = 0.039 [109], and 6.1 ± 1.28 to 1.15 ± 1.0; *p* < 0.01 [110]. The random effect meta-analysis of the currently available results shows a statistically significant difference in favor of the treatment (*p* < 0.001). On the basis of these results, in cases of mild OSA, a 12-week treatment with ICS should be considered.

Finally, the two SR by Zhang et al. [107] and Chada et al. [108] respectively of low and moderate quality, concluded that ICS can significantly improve nasal obstruction symptoms in children with moderate and severe AH while reducing t adenoids size. The treatment seemed to be safe and few adverse events have been reported. Long-term efficacy has not been assessed.

Two more recent RCTs reported a significant effect of ICS on the reduction of adenoid tissue and the degree of hypertrophy [111, 112]. Finally, in a non-randomized study, 35 children who had undergone adenoidectomy were treated with MF for 6 months and starting 3 weeks after wound healing compared to 35 children undertaking local saline solution [113]. At the end of the follow up the degree of hypertrophy in the treated group was significantly lower than in controls (1.20 ± 0.41 vs 2.20 ± 0.88; *p* = 0.001). Also score of the symptoms was significantly lower in treated children, even if this effect was not clinically relevant (on average 0.24 vs 0.94; p = 0.001).

Two other studies considered the reduction of adenoidectomies as marker of ICS efficacy. The first study [114] included in the SR of Chohan et al. enrolled 65 non-atopic patients aged 5–15 years being symptomatic for at least 6 months, with 75% occlusion of the nasopharynx (endoscopic diagnosis). The intervention group of 35 children was treated with 100 μg of intranasal MF per day for 12 weeks. The control group received NaCl 0.9% solution. Four 4 months later, 77.2% of subjects from the treated group were excluded from the surgery list, while all the non-treated subjects underwent surgery. The second study [115], is a double-blind trial which after the first 2 weeks shifted to an open study for 12 weeks. Fifty children aged 6–8 years, with AH grade > 2 and AHI > = 2/h, were treated with budesonide and 50 children used physiological solution. At the end of the 12 weeks of follow-up, 24 children of the treated group and 33 children of the untreated group underwent adenoidectomy; RAR = 22% (3.6–40.4%). In both studies a trend in favor of treatment was observed even if no statistically significant difference was found from the random effect meta-analysis of the currently available results (RR = 0.43 95% CI = 0.14–1.31; *p* = 0.14). In conclusion, no SR considered the reduction of the number of adenoidectomies as an outcome. Only 2 studies, while not achieving statistical significance, were consistently supporting the use of ICS. Considering the relevant benefits (reduction of adenoidectomies) and the negligible risks (sustainable cost, high compliance, few and transient adverse events), a trial with ICS should still be recommended in children who are ongoing surgery.

Limited evidences suggest that, in some cases, surgery might be avoided by prolonged ICS treatment (12 weeks), even if no long-term follow-up data are available. Children who benefit from this approach are those showing a good clinical response immediately after 2 weeks of treatment [116].

Nasal obstruction symptoms were assessed by three SR [106–108]: all studies showed overlapping results confirming the benefit of treatment with endonasal ICS for 8–12 weeks; however, the statistical significance decreased when only blinded studies are analyzed. For this reason, only 2 blind studies published after the SR were considered.

In the study by Hong et al., [115] all children in the treatment group showed a significant reduction in the nasal obstruction index (NOI) compared to controls. The significant difference of NOI between the two groups of children was confirmed after 12 weeks of open treatment (*p* < 0.001).

Liu et al. [117] investigated children with AH and associated perennial allergic rhinitis. Initially, the children were divided into 2 groups (MF versus placebo). After the first 6 weeks of treatment, the group treated with MF showed a significant reduction in the symptom score (6.9 ± 1.5 vs 16.5 ± 1.3, *p* < 0.05) compared to placebo, but 2 weeks later the score was at the baseline again. In the second phase of treatment, following a 2-week wash-out period, the non-responders were divided into 4 groups: Oxymetazoline (OXY) and placebo, MF and placebo, MF and OXY, placebo and placebo for 8 weeks. The subjective and total symptom scores in the MF/OXY group were significantly lower compared to other groups (p < 0.05) [117]. In conclusion, most of evidences demonstrate a significant reduction in nasal obstruction symptoms as well as in the size of the adenoids, although further studies are needed to evaluate the most effective molecule, the ideal duration of treatment and the presence of factors influencing the outcomes, such as allergic rhinitis.

As far as the adverse events occurring during ICS treatment, Chadha et al. [108] reported mild local reaction such as burning sensation and nosebleeds described in 2 studies [116, 118]. The reported adverse events did not cause drop out. Three studies, selected in the review by Chohan et al. [106] reported epistaxis [119–121], without significant differences between the group treated with MF and the group treated with placebo.

Also in the study by Hong et al. [115] no significant difference was reported between the group treated with budesonide for 12 weeks and the control group; moreover, nose bleeding improved after correcting administration technique in all reported cases.

20. In children with AH and OME, ICS are not recommended to treat OME (Very low quality evidence. Weak negative recommendation).

GL on OME published in 2016 [104], with good methodological quality, in the 8a statement reported a strong negative recommendation on the use of ICS for the treatment of OME in children aged from 2 months to 12 years. Patients treated with ICS for the primary management of another coexisting condition, were excluded. Moreover, in oral and topical steroids section, the authors suggested that topical nasal steroids might be responsible of short-term benefit in children with AH, but the extent of this effect was small and the dosage reported in one study was higher than recommended. Therefore, they did not formulate recommendations for this condition.

The SR by Chohan et al. [106] reported the results of 2 studies evaluating the efficacy of Mometasone fuorate [120, 121]. Even if in each single study the reduction of the OME seems to be statistically significant, the random effect meta-analysis, which takes into account the heterogeneity of the studies, does not confirm this result (RR = 0.33 [0.05, 2.41].

In the study by Hong et al. [115] the outcomes of OME were similar between the patients treated with budesonide for 12 weeks and controls (RR = 0.80 [0.40, 1.60]).

### Laryngotracheitis and laryngospasm

Laryngospasm (croup) is defined as “a generally nocturnal episode with sudden onset, sometimes preceded by 24-72 hours of non-specific cough, runny nose and fever, manifesting itself with barking cough, hoarseness and inspiratory stridor”. Viral croup is the second most frequent cause of respiratory distress in children [122]. The estimated incidence is around 3% of children per year; less than 3% of them are hospitalized and 1–2% need oral intubation treatment [123], with a probability of death of around 1 in 30,000 cases [124].

Viral croup is caused by an obstructive airway event characterized by a prevalent involvement of the subglottic area of ​​the larynx [122, 125, 126], so that the terms v*iral croup, laryngitis, laryngotracheitis, laryngotracheobronchitis* and *laryngospasm* are commonly considered as synonyms indicating the same involvement of the subglottic area [127]. However, many authors, suggest to differently define the clinical conditions depending on the presence or absence of non-specific warning signs and inspiratory stridor, as follow: “*Spasmodic croup*” (or laryngospasm or spasmodic laryngitis) when no warning symptoms nor fever but stridor is present; “*Laryngotracheitis*”when warning symptoms are present but stridor is missing [128].

More recently Kligman et al. [127] suggested to distinguish 4 conditions:

*1. Croup (Laryngotracheobronchitis), 2. Acute Epiglottitis (Supraglottitis), 3. Acute Infectious Laryngitis, and 4. Spasmodic Croup,* wherethe terms *Croup, Laryngitis* and *Spasmodic Croup* identify infectious and non-infectious diseases with laryngeal involvement.

The most common causative viruses are Parainfluenza viruses, other viruses (Influenza A and B, Respiratory Syncytial Virus, Rhinovirus, Coronavirus, Metapneumovirus and Adenovirus) and rarely *Mycoplasma pneumoniae* [128, 129]. The diagnosis is usually easy, both in outpatient settings and in emergency settings and the Westley scoring scale allows to assess croup severity [130].

Treatment includes nebulized adrenaline, systemic and inhaled corticosteroids. High-dose inhaled steroids are believed to have a faster anti-oedema effect (“membrane” or “non-genomic” effect), compared to oral corticosteroids. This effect seems related to the ability of steroids to bind to an endocellular receptor able to determine an increase of smooth muscle tone of the laryngeal and bronchial vessels, with consequent vasoconstriction and reduction of local oedema (“bleaching effect”) [131].

#### Is the use of the ICS indicated in the treatment of laringotracheitis (acute infectious laryngitis)?

**Is the use of the ICS indicated in the treatment of laryngospasm (croup)?**

To answer the questions consensus panel considered guidelines, systematic reviews and primary studies on children aged 0 to 18 years presenting with laryngospasm, croup, laryngotracheitis, laryngotracheobronchitis both hospitalized or admitted as outpatients in primary care settings or emergency departments.

No evidence was found on the efficacy and safety of ICS in viral laryngotracheitis.

As for evidence-based GL, most of the documents are intra-hospital or Regional Health Authority consensus documents that do not meet the minimum methodological criteria required to be defined as GL. The only valid GL, produced by Children’s Mercy Hospital [132], does not answer to any clinical questions taken into consideration (such as the use of ICS in croup). Two RS were also included [133, 134].

In order to formulate the recommendations, the critical outcomes considered were the change in croup severity score at the shortest follow-up (2 or 6 h) and the need for other treatments, specifically intubation and tracheotomy. Moreover, the proportion of additional visits/admissions and the length of stay in the emergency department or in the hospital were also considered.

The analyzed comparisons were: ICS versus placebo to evaluate ICS efficacy; ICS versus CSO and ICS versus adrenaline to evaluate ICS efficacy compared to other treatments, and the association dexamethasone-budesonide versus dexamethasone alone, to evaluate whether adding ICS to oral therapy improves short- and medium-term outcomes.

Data were extracted and meta-analyzes were recalculated for some comparisons more specifically relevant to the questions and to the outcomes considered for this document.

##### Recommendations

21. Children with viral laryngotracheitis should not be treated with ICS. (Expert opinion. Weak negative recommendation).

There is no evidence on the use of ICS in laryngotracheitis, while currently available evidence regarding treatment of laryngospasm (croup), is mainly of low quality because of small studies hampered by severe bias

22. In children with mild or moderate croup, ICS, specifically high-dose nebulized budesonide, can be administered. (Low quality evidence. Weak positive recommendation).

Griffin S. et al. conducted a SR including 8 RCTs, and evaluated the efficacy of nebulized corticosteroids versus placebo [133] in children presenting with croup. Concerning primary outcomes, patients treated with ICS showed improvement in croup score at 5 h from ICS treatment (RR = 1.48, 95% CI = 1.27–1.74) and a reduction in the hospitalization rate (RR = 0.56, 95% CI = 0.42–0.75). The NNT (i.e. the proportion of children with moderate or severe croup to be treated with ICS in the emergency department in order to avoid one hospitalization) ranged from 2.9 to 8.4 in the studies included in the SR. These results were confirmed by a recent high quality SR [135]. Gates et al. included 43 RCTs published up to April 2018, with a total of 4565 patients aged 0–18 years. Beclomethasone, betamethasone, budesonide, dexamethasone, fluticasone and prednisolone were evaluated and compared to placebo, adrenaline, or between each other also including different inhalation techniques and dosages. Primary outcomes were the variation of croup severity score and the rate of access to the emergency department and hospitalizations. Secondary outcomes were the length of stay in the emergency department or in the hospital, the clinical improvement at 2, 6 and 12 h from treatment and the use of additional treatments. Concerning Budesonide vs placebo, 4 studies [135–138] with an overall low quality of the evidence have been included. Nebulized budesonide (2 mg/4 ml) compared to placebo was more effective in reducing symptoms severity in 2 h in children with moderate to severe croup (SM = − 1.01; − 1.71 - -0.3, *p* = 0.005. Low quality evidence, large effect size). Also with regard to the outcome of readmission to hospital, budesonide was more effective than placebo (RR = 0.42; 0.19–0.90, *p* = 0.025). No adverse effects were reported.

23. In children with moderate to severe croup, corticosteroids should be preferably administered systemically (Moderate quality evidence. Weak positive recommendation).

*Budesonide* versus *oral dexamethasone.* A SR analyzed 4 RCTs for a total 326 children [134]. Compared to oral dexamethasone, nebulized budesonide led to a significant increase in the croup score after six hours from administration [Standardized Mean Difference (SMD) 0.46, 95% CI = 0.79 to 0.13; *p* = 0.006; moderate quality evidence, moderate effect size]. Authors explained these results as due to the easier and faster oral administration in children. No significant difference was found in the rate of further visits or hospitalizations between the children treated with dexamethasone and those treated with budesonide, nor in the length of stay in the emergency department or hospital (low quality evidence).

*Beclomethasone* versus *dexamethasone.* Gates et al. reported only one RCT of 39 children and comparing dexamethasone (0,6 mg/Kg im) to beclomethasone (200 μg/dose administered via pMDI + spacer. The only outcome assessed was reassessment in the emergency department or hospitalizations, without significant difference among study groups (RR = 0.0; 95% CI = − 0.09, 0.09; *p* = 1.0) [134].

The only RCT published after the SR by Gates et al. was not included because the full text is in Chinese [139]. Nevertheless, the results reported in the abstract confirmed that ICS administration (budesonide 1 mg every 30 min two times and then every 12 h) did not improve the clinical scores at 12 and 24 h compared to controls who were taking dexamethasone (0.3–0.5 mg/kg intramuscle).

24. In severe croup, it is not recommended to use nebulized budesonide to replace nebulized adrenaline (Low quality evidence. Strong negative recommendation).

*Budesonide and beclomethasone* versus *nebulized adrenaline.* Gates et al. analyzed 2 RCTs comparing adrenaline to ICS (1 versus nebulized budesonide, 1 versus nebulized beclomethasone) for a total of 130 children. Compared to adrenaline, ICS treatment did not significantly improve the croup score after 2 h from administration (SMD = 0.77, 95% CI = − 0.24 at 1.77; *p* = 0.13; low quality evidence; Analysis 2.1). No difference among study groups was found also after 6, 12 or 24 h. Moreover, in a subgroups analysis of 33 children treated with beclomethasone, Gates et al. observed that adrenaline was significantly more effective in reducing croup score (SMD = 1.41, 95% CI from 0.62 to 2.19; *p* < 0.001). Authors confirmed similar results also in another subgroup analysis of 31 children treated with dexamethasone (SMD = 1.13, 95% CI from 0.35 to 1.91; *p* = 0.005). The comparison between budesonide as well as dexamethasone vs nebulized adrenaline at 12 and 24 h did not show significant difference in any subgroups. Regarding further hospital admissions or additional treatments such as intubation, no significant difference was reported between the study groups (Moderate quality evidence).

Only one RCT on 66 children, showed no difference in the score reduction between *budesonide and adrenaline* (SMD = 0.26, 95% CI = − 0.22 to 0.75; *P* = 0.29). In conclusion, there are no evidences of greater effectiveness of budesonide compared to nebulized adrenaline: therefore, actual data are insufficient to recommend nebulized budesonide to replace adrenaline for the treatment of severe croup [134].

25. In children with moderate or severe croup, it is not recommended to combine nebulized budesonide and SC (High quality evidence. Strong negative recommendation).

*Budesonide and dexamethasone* versus *dexamethasone.* 3 RCTs for a total of 255 children, compared the association of nebulized budesonide (2 mg in 4 ml, single dose) with oral dexamethasone (single dose of 0,6 mg/Kg) vs oral dexamethasone monotherapy. No significant difference between the two treatments regimens was found when considering the reduction of croup score after 6 h from treatment administration (High quality evidence). The clinical score was also similar both when considering the rate of further emergency department evaluations and the rate of hospitalizations (high quality evidence). Therefore, the association between oral dexamethasone and budesonide does not seem to provide additional benefits to the administration of dexamethasone alone [134].

26. In children with croup, treatment with fluticasone or beclomethasone (pMDI + spacer) is not recommended. (Very low quality of evidence. Weak negative recommendation). There is no evidence on other ICS nor on administration techniques other than nebulization: therefore, their use is not recommended (Expert opinion. Weak negative recommendation).

From the results of a single study (very low quality evidence), fluticasone (4 puffs, each 250 μg, administered in 30 min) was not effective in reducing the severity score (1 RCT, 17 children; SMD = + 0.45; SD = from − 0.52 to + 1.42; *p* = 0.36) and length of stay in the emergency department (MD = + 4.8 h [from − 12.34 to + 21.94]; *p* = 0.58) [134].

## Data Availability

Not applicable.

## Abbreviations

AAAAI: American Academy of Allergy, Asthma, and Immunology
AAP: American Academy of Pediatrics
ABRS: Acute Bacterial Rhinosinusitis
ACAAI: American College of Allergy, Asthma, and Immunology
ACTH: AdenoCorticoTropic Hormone
ADRs: Adverse Drug Reactions
AGREE: Appraisal of Guidelines, Research and Evaluation
AH: Antihistaminic
AHI: Apnea Hypopnea Index
AIFA: Agenzia Italiana del Farmaco (Italian Medicines Agency)
AMSTAR: Assessment of multiple systematic reviews
AR: Allergic rhinitis
ARIA: Allergic Rhinitis and its Impact on Asthma
ARS: Acute RhinoSinusitis
BSACI: British Society for Allergy & Clinical Immunology
BTS: British Thoracic Society
CDSR: Cochrane Database of Systematic Reviews
CFC: 19 / 5000 Chlorofluorocarbons
CI: Confidence interval
CRH: Corticotropin-Releasing Hormone
CRS: Chronic RhinoSinusitis
CRSsP: Chronic Rhinosinusitis With Nasal Polyps
CRSwNP: Chronic Rhinosinusitis without Nasal Polyps
CS: Corticosteroids
CSI-IND: Corticosteroids Inhalant-Indacaterolo
CT: Computed Tomography
DARE: Database of Abstract of Review of Effects
DPI: Dry-Powder Inhaler
EBM: Evidence Based Medicine
ED: Emergency Department
EMA: European Medicine Agency
EPAG: European Pharmaceutical Aerosol Group
EPOS: European Position Paper on Rhinosinusitis and Nasal Polyps
EVW: Episodic Viral Wheezing
FEV1: Forced Expiratory Volume in the 1st second
FIMP: Federazione Italiana Medici Pediatri (Italian Federation of Pediatricians)
GH: Growth Hormone
GILZ: Glucocorticoid-Induced Leucine Zipper
GINA: Global Initiative for Asthma
GL: Guideline
GR: Glucocorticoid Receptors
GRADE: Grading of Recommendations Assessment, Development and Evaluation
GRE: Glucocorticoid Responsive Elements
HFA: Hydrofluoroalkanes
HPA: Hypothalamic–pituitary–adrenal axis
AH: Adenoid hypertrophy
ICAR:AR: International Consensus Statement on Allergy and Rhinology: Allergic Rhinitis
ICS: Inhaled Corticosteroids
INAH: IntraNasal Antihistamines
IND: IntraNasal Decongestant
IPB: Ipratropium Bromide
KGF: Keratinocyte Growth Factor
LABA: Long Acting Beta-Agonist
LAR: Local Allergic Rhinitis
LoE: Levels of Evidence
LTRA: Leukotriene Receptor Antagonist
M.A.L.T.: Mucosal-Associated Lymphoid Tissue
MAPK: Mitogen Activated Protein Kinase
MD: Mean Deviation
MDI: Metered Dose Inhalers
MF: Mometasone Furoate
MFNS: Mometasone Furoate Nasal Spray
MMAD: Mass Median Aerodynamic Diameter
MRT: Maintenance and Reliever Therapy
MTW: Multiple Trigger Wheezing
N.A.L.T.: Nasal-Associated Lymphoid Tissue
NAPT: Nasal Provocation Test
NAR: Non-allergic rhinitis
NICE: The National Institute for Health and Care Excellence
NNT: Number-Needed-to-Treat
NOI: Nasal Obstruction Index
NZ: New Zealand
OAH: Oral Antihistamines
OCS: Oral Corticosteroids
OME: Effusive Otitis Media
OSAS: Obstructive Sleep Apnea Syndrome
OXY: Oxymetazolin
PAR: Perennial Allergic Rhinitis
PCRS: Pediatric Chronic RhinoSinusitis
PEF: Peak of Expiratory Flow
PICOs: Population, Intervention, Comparison, Outcome
pMDI: Pressurised Metered Dose Inhalers
PNLG: Programma Nazionale per le Linee Guida (National Program for Guidelines)
QoL: Quality of Life
RCT: Randomized Control Trial
REM: Rapid Eye Movements
RMP: Risk Management Plan
RNF: Rete Nazionale Farmacovigilanza (National Pharmacovigilance Network)
RR: Relative Risk
RS: Rhinosinusitis
SABA: Short Acting Beta-Agonist
SAR: Seasonal Allergic Rhinitis
SCS: Systemic Corticosteroids
SIDRIA: Studi Italiani sui Disturbi Respiratori nell'Infanzia e l’Ambiente (Italian Studies on Childhood Respiratory Disorders and the Environment)
SIGN: Scottish Intercollegiate Guidelines Network
SMD: Standardized Mean Difference
SR: Systematic Reviews
STICS: Step-up Yellow Zone Inhaled Corticosteroids
TNSS: Total Nasal Symptom Score
TOSS: Total Ocular Symptom Score
VAS: Visual analog scale
