# Correction: Inter-society consensus for the use of inhaled corticosteroids in infants, children and adolescents with airway diseases

**DOI:** 10.1186/s13052-022-01231-8

**Published:** 2022-02-25

**Authors:** Marzia Duse, Francesca Santamaria, Maria Carmen Verga, Marcello Bergamini, Giovanni Simeone, Lucia Leonardi, Giovanna Tezza, Annamaria Bianchi, Annalisa Capuano, Fabio Cardinale, Giovanni Cerimoniale, Massimo Landi, Monica Malventano, Mariangela Tosca, Attilio Varricchio, Anna Maria Zicari, Carlo Alfaro, Salvatore Barberi, Paolo Becherucci, Roberto Bernardini, Paolo Biasci, Carlo Caffarelli, Valeria Caldarelli, Carlo Capristo, Serenella Castronuovo, Elena Chiappini, Renato Cutrera, Giovanna De Castro, Luca De Franciscis, Fabio Decimo, Iride Dello Iacono, Lucia Diaferio, Maria Elisa Di Cicco, Caterina Di Mauro, Cristina Di Mauro, Dora Di Mauro, Francesco Di Mauro, Gabriella Di Mauro, Mattia Doria, Raffaele Falsaperla, Valentina Ferraro, Vassilios Fanos, Elena Galli, Daniele Giovanni Ghiglioni, Luciana Indinnimeo, Ahmad Kantar, Adima Lamborghini, Amelia Licari, Riccardo Lubrano, Stefano Luciani, Francesco Macrì, Gianluigi Marseglia, Alberto Giuseppe Martelli, Luigi Masini, Fabio Midulla, Domenico Minasi, Vito Leonardo Miniello, Michele Miraglia Del Giudice, Sergio Renzo Morandini, Germana Nardini, Agostino Nocerino, Elio Novembre, Giovanni Battista Pajno, Francesco Paravati, Giorgio Piacentini, Cristina Piersantelli, Gabriella Pozzobon, Giampaolo Ricci, Valter Spanevello, Renato Turra, Stefania Zanconato, Melissa Borrelli, Alberto Villani, Giovanni Corsello, Giuseppe Di Mauro, Diego Peroni

**Affiliations:** 1 Department of Pediatrics, Policlinico Umberto I, Sapienza University of Rome, Rome, Italy; 2 Department of Translational Medical Sciences, Federico II University, Naples, Italy; 3 Family Pediatrician Local Health Unit Salerno, Vietri sul Mare, Italy; 4 Family Pediatrician, Local Health Unit, Ferrara, Italy; 5 Family Pediatrician, Local Health Unit, Mesagne, Italy; 6 Maternal, Infantile and Urological Sciences Department, Sapienza University, Rome, Italy; 7 Pediatric Department, Franz Tappeiner Hospital, Meran, Italy; 8 Pediatric Unit, Department of Women’s and Children’s Health, San Camillo Forlanini Hospital, Rome, Italy; 9 Department of Experimental Medicine, University “Luigi Vanvitelli”, Regional Centre of Pharmacovigilance Campania, Naples, Italy; 10 Pediatric and Emergency Unit Giovanni XXIII Pediatric Hospital University of Bari, Bari, Italy; 11 Family Pediatrician Local Health Unit Latina, Latina, Italy; 12 Family Pediatrician Local Health Unit, Turin and IRIB-CNR, Palermo, Italy; 13 Family Pediatrician Local Health Unit, Ferrara, Italy; 14 Allergy Centre, Department of Pediatric Sciences IRCCS Gaslini Institute, Genoa, Italy; 15 Departmental Operative Unit of Diagnostic and Surgical Videoendoscopy of the Upper Airways, Asl Napoli 1 Center, Naples, Italy; 16 Maternal, infantile and urological sciences Department, Pediatric Allergic Unit, Sapienza University, Rome, Italy; 17 Paediatrics Unit, Reunited Hospitals Castellammare of Stabia, Naples, Italy; 18 Paediatric Unit, ASST-Rhodense, RHO, Milan, Italy; 19 Family Pediatrician, Local Health Unit Tuscany center, Florence, Italy; 20 Pediatric Unit San Giuseppe Hospital, Empoli, Florence, Italy; 21 Family Paediatrician, Local Health Unit, FIMP National President, Livorno, Italy; 22 Department of Obstetrics Gynaecology and Pediatrics, Azienda USL-IRCCS di Reggio Emilia, Reggio Emilia, Italy; 23 Pediatric Unit, Department of Mother and Child, AUSL-IRCCS, Reggio Emilia, Italy; 24 Department of Woman, Child and of General and Specialized Surgery, University “Luigi Vanvitelli”, Naples, Italy; 25 Family Paediatrician Local Health Unit Nettuno-Anzio, Rome, Italy; 26 Paediatric Infectious Disease Unit, Meyer Children’s University Hospital, Department Of Health Sciences, University of Florence, Florence, Italy; 27 Pediatric Pulmonology Unit, Academic Department of Paediatrics, Bambino Gesù Children’s Hospital, IRCCS, Rome, Italy; 28 Endocrinologist Specialist, Salerno, Italy; 29 Independent Paediatrician Researcher, Benevento, Italy; 30 Department of Paediatrics, Aldo Moro University of Bari, Bari, Italy; 31 Paediatrics Unit, University Hospital of Pisa, Department of Clinical and Experimental Medicine, University of Pisa, Pisa, Italy; 32 General Paediatrics and Paediatric Acute and Emergency Unit, University Hospital San Marco, University of Catania, Catania, Italy; 33 Family Paediatrician Local Health Unit, Ausl, Modena, Italy; 34 Family Paediatrician, Local Health Unit, Caserta, Italy; 35 Primary Care Paediatrician, Local Health Unit, National Secretary for the Scientific and Ethical Activities of FIMP, Chioggia, Italy; 36 Neonatal Intensive Care Unit and Neonatal Accompaniment Unit, University Hospital San Marco, University of Catania, Catania, Italy; 37 Unit of Paediatric Allergy and Respiratory Medicine Women’s and Children’s Health Department, University Hospital Padua, Padua, Italy; 38 Neonatal Intensive Care Unit, Neonatal Pathology and Neonatal Section, AOU and University of Cagliari, Monserrato, CA Italy; 39 Pediatric Allergy Unit, Department of Paediatric Medicine, S. Pietro Hospital Fatebenefratelli, Rome, Italy; 40 Foundation IRCCS Ca’ Granda Ospedale Maggiore Policlinico di Milano, UOSD Paediatric Highly Intensive Care Unit, Milan, Italy; 41 Pediatric Asthma and Cough Center Istituti Ospedalieri Bergamaschi, Gruppo Ospedaliero San Donato, Ponte San Pietro, Bergamo, Italy; 42 Family Pediatrician Local Health Unit Teramo, Teramo, Italy; 43 Paediatric and Neonatology Unit Santa Maria Goretti Hospital, Department of Pediatrics, University of Pavia, Pavia, Italy; 44 Paediatric and Neonatology Unit Santa Maria Goretti Hospital, Department of Pediatrics, Sapienza University, Rome, Italy; 45 Pediatric and Neonatal Intensive Care Unit Fatebenefratelli Isola Tiberina, Rome, Italy; 46 Allergist Pediatrician National Secretary of Italian Federation for Medical Scientific Societies (FISM), Rome, Italy; 47 Department of Pediatrics G. Salvini Hospital Garbagnate Milanese, Milan, Italy; 48 Pediatric Pulmonology and Subintensive Respiratory Therapy Unit Department of Pediatrics Santobono-Pausilipon Children’s Hospital, Naples, Italy; 49 Pediatric Unit Great Metropolitan Hospital Reggio Calabria, Reggio Calabria, Italy; 50 Department of Biomedical Science and Human Oncology, University of Bari, Children’s Hospital “Giovanni XXIII”, Bari, Italy; 51 Family Pediatrician, Local Health Unit, Latina, Italy; 52 Department of Translational Medical Sciences, Pediatric Pulmonology, Federico II University, Naples, Italy; 53 Division of Pediatrics, University Hospital of Udine, Udine, Italy; 54 Department Health Science, University of Florence, Florence, Italy; 55 Department of Human Pathology in Adult and Development Age, Pediatric Unit, University of Messina, Messina, Italy; 56 Pediatric Unit, S. Giovanni di Dio Hospital, Crotone, Italy; 57 Paediatric Section Department of Surgery, Dentistry, Paediatrics and Gynaecology University of Verona, Verona, Italy; 58 Family Pediatrician, Paediatric Allergy, Local Health Unit TO1, Turin, Italy; 59 Department of Pediatrics, IRCC San Raffaele, Milan, Italy; 60 Alma Mater Studiorum, Univesity of Bologna, Bologna, Italy; 61 Family Pediatrician Local Health Unit, Caselle Torinese, Vicenza, Italy; 62 Family Pediatrician Local Health Unit Caselle Torinese, Turin, Italy; 63 Department of Translational Medical Sciences, Federico II University, Naples, Italy; 64 Unit of Pediatric Allergy and Respiratory Medicine Women’s and Children’s Health Department University Hospital, Padua, Italy; 65 Family Pediatrician, Local Health Unit Salerno, Aversa, Italy; 66 Department of Clinical and Experimental Medicine, Section of Pediatrics, University of Pisa, Pisa, Italy

**Correction to: Italian Journal of Pediatrics 47, 1-24 (2021)**

**https://doi.org/10.1186/s13052-021-01013-8**

The original article contained an incorrect institution for Affiliation #22 which has since been corrected.

